# Single‐Nucleus RNA Sequencing Reveals Mid‐Gestational Neurodevelopment Features in the Superior Temporal Plane from Fetuses with Nonsyndromic Cleft Lip and Palate

**DOI:** 10.1002/advs.202504191

**Published:** 2025-10-21

**Authors:** Liu‐Lin Xiong, Xin‐Wei Huang, Qiu‐Xia Xiao, Ying‐Gang Zheng, Hao‐Yue Qin, Chang‐Le Fang, Li Chen, Ruo‐Lan Du, Qiu‐Lin Wang, Li‐Ren Huangfu, Ye‐Qing Fei, Yang‐Yang Zhao, Chen‐Yang Zhai, Ze‐Mian Chen, Shi‐Kun Yuan, Yu‐Ji Zhou, Xing‐Man He, Ting‐Ting Zhi, Xiao‐He Tian, Ting‐Hua Wang

**Affiliations:** ^1^ Institute of Neuroscience Kunming Medical University Kunming 650500 China; ^2^ Department of Anesthesiology The Third Affiliated Hospital of Zunyi Medical University (The First People’s Hospital of Zunyi) Zunyi China; ^3^ Translational Neuromedicine Laboratory The Affiliated Hospital of Zunyi Medical University Zunyi 563000 China; ^4^ Shanghai Key Laboratory of Anesthesiology and Brain Functional Modulation Clinical Research Center for Anesthesiology and Perioperative Medicine Translational Research Institute of Brain and Brain‐Like Intelligence Shanghai Fourth People's Hospital, Tongji University Shanghai 200434 China; ^5^ Institute of Neurological Disease, National‐Local Joint Engineering Research Center of Translational Medicine, West China Hospital Sichuan University Chengdu 610041 China; ^6^ Department of Family Planning The Affiliated Hospital of Zunyi Medical University Zunyi 563000 China; ^7^ School of Anesthesiology Zunyi Medical University Zunyi 563000 China; ^8^ The First Clinical Institute Zunyi Medical University Zunyi 563000 China

**Keywords:** MEF2C, neurodevelopmental impairments, nonsyndromic cleft lip and palate, superior temporal plane, synaptic function

## Abstract

Nonsyndromic cleft lip and palate (NSCLP) is a common craniofacial malformation increasingly recognized to involve neurodevelopmental abnormalities, though the molecular basis remains unclear. Here, single‐nucleus RNA sequencing of the superior temporal plane from mid‐gestation NSCLP fetuses is performed, and profound alterations in cell‐type composition, intercellular communication, and transcriptional programs are uncovered. Integrative analyses with weighted gene co‐expression network analysis and single‐cell regulatory network interference and clustering based on single‐nucleus transcriptomes identify myocyte enhancer factor 2C (MEF2C) as a shared transcriptional regulator consistently downregulated in excitatory and inhibitory neurons across mid‐term gestation, which is validated in NSCLP fetal brain tissues. MEF2C expression is negatively correlated with synaptophysin immunofluorescence intensity. In MEF2C‐deficient primary cortical neurons, impaired synaptic formation, reduced postsynaptic density protein‐95 expression, and weakened excitatory postsynaptic transmission without altering intrinsic excitability are found. Upstream regulators of MEF2C are enriched for pathways controlling neuronal differentiation, synaptic plasticity, and epigenetic regulation, suggesting broad disruption of neurodevelopmental programs. Together, this study provides molecular evidence of disrupted brain development in NSCLP and implicates MEF2C as a potential mediator of neurodevelopmental impairments.

## Graphical abstract

Single‐nucleus RNA sequencing of mid‐gestation brains from fetuses with nonsyndromic cleft lip and palate reveals major disruptions in cell composition, cell‐to‐cell signaling, and gene regulation. The transcription factor MEF2C is identified as a central regulator of these changes and shows that lowering MEF2C impairs synapse formation, linking cleft lip and palate to abnormal brain development.

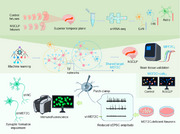

## 1 Introduction

Cleft lip and palate (CLP) is a common craniofacial malformation that occurs in approximately 1 in 700 live births globally.^[^ ^1^ ^]^ The incidence is notably higher in East Asian populations, particularly in China and Japan.^[^ ^2^ ^]^ CLP is categorized into two types: syndromic cleft lip and palate and nonsyndromic cleft lip and palate (NSCLP), depending on the presence of additional systemic malformations. NSCLP accounts for ≈70% of CLP cases according to statistics from the International Cleft Lip and Palate Registry.^[^ ^3^ ^]^ Individuals afflicted with NSCLP frequently encounter a plethora of challenges. These challenges encompass speech dysfunction, neurocognitive developmental delay, and social adjustment.^[^ ^1^ ^]^ Current research predominantly focuses on surgical repair techniques for the maxillofacial clefts, while the molecular mechanisms underlying the neurodevelopmental abnormalities in NSCLP remain to be further elucidated.^[^ ^4^ ^]^ Genetic factors play crucial roles in the embryonic maxillofacial fusion process. It is imperative to develop a comprehensive framework integrating multi‐omics analyses, including epigenetic regulation, neural circuit formation, and glial cell interactions. New research suggests that neurodevelopmental abnormalities in NSCLP coexist with maxillofacial malformations and can lead to cognitive deficits such as central auditory processing deficits, language deficits, learning disabilities, and attention deficits.^[^ ^5^, ^6^ ^]^ It is noteworthy that NSCLP neonates exhibit abnormal auditory event‐related potentials, suggesting that brain structural and functional changes may originate in fetal stages.^[^ ^5^, ^7^ ^]^ Recent studies have identified structural differences in the superior temporal plane (STP)—a key region for auditory and language processing—in NSCLP patients, including variations in cortical thickness and volume.^[^ ^8^, ^9^ ^]^ Furthermore, functional magnetic resonance imaging (fMRI) studies have provided additional evidence that NSCLC patients have associated cortical thickness abnormalities. This highlights the need for further detailed studies at the cellular and molecular levels.^[^ ^9^ ^]^ Nevertheless, the precise mechanisms underpinning these neurodevelopmental anomalies remain unclear.

High‐throughput single‐nucleus RNA sequencing (snRNA‐seq) has revolutionized the entire field of biomedical research, with human and mouse cell atlases constructed through sc/snRNA‐seq platforms in the last few years.^[^ ^10^, ^11^ ^]^ This innovative platform has elucidated cell‐type–specific vulnerability patterns in various neuropathological conditions, such as Alzheimer's disease (entorhinal cortex L2/3 neurons),^[^ ^12^ ^]^ major depressive disorder (astrocyte subcluster MDD‐AC3)^[^ ^13^ ^]^ and neurodevelopmental disorders (including Rett syndrome (MECP2+ GABAergic interneurons) and autism spectrum disorder (ASD‐associated microglial states)).^[^ ^14^ ^]^ Deep learning presents as a means of simultaneously extracting and integrating features from multiple data types to predict the efficacy of drug pairs.^[^ ^15^, ^16^ ^]^ The recent advances in deep learning may open a new chapter in the search for computational drug response prediction models and ultimately result in more accurate tools for therapy response.^[^ ^16^ ^]^ The convergence of these technologies, which includes the application of snRNA‐seq for the mapping of NSCLP cortical mosaicism and the utilization of machine learning for the modelling of gene‐glia interaction dynamics, has led to the identification of dysregulated Wnt/β‐catenin signaling in NSCLP glutamatergic progenitors. This identification establishes a framework for the analysis of neurodevelopmental trajectories that are perturbed in cases of craniofacial malformations.^[^ ^17^, ^18^ ^]^

In this study, we performed snRNA‐seq of the STP region from normal and NSCLP fetal brains across mid‐term developmental stages, aiming to delineate the cellular and molecular landscape of NSCLP‐associated brain abnormalities. By integrating machine learning with transcriptomic profiling, we identified key transcriptional regulators and disrupted signaling networks that may contribute to the cognitive dysfunction observed in NSCLP. These findings provide molecular evidence for neurodevelopmental abnormalities in NSCLP and establish a framework for future mechanistic studies. Importantly, this work lays the foundation for developing targeted therapeutic strategies to mitigate cognitive impairments associated with NSCLP.

## 2 Results

### 2.1 STP Single‐Cell Transcriptome in the Fetuses with NSCLP

**Figure 1:**
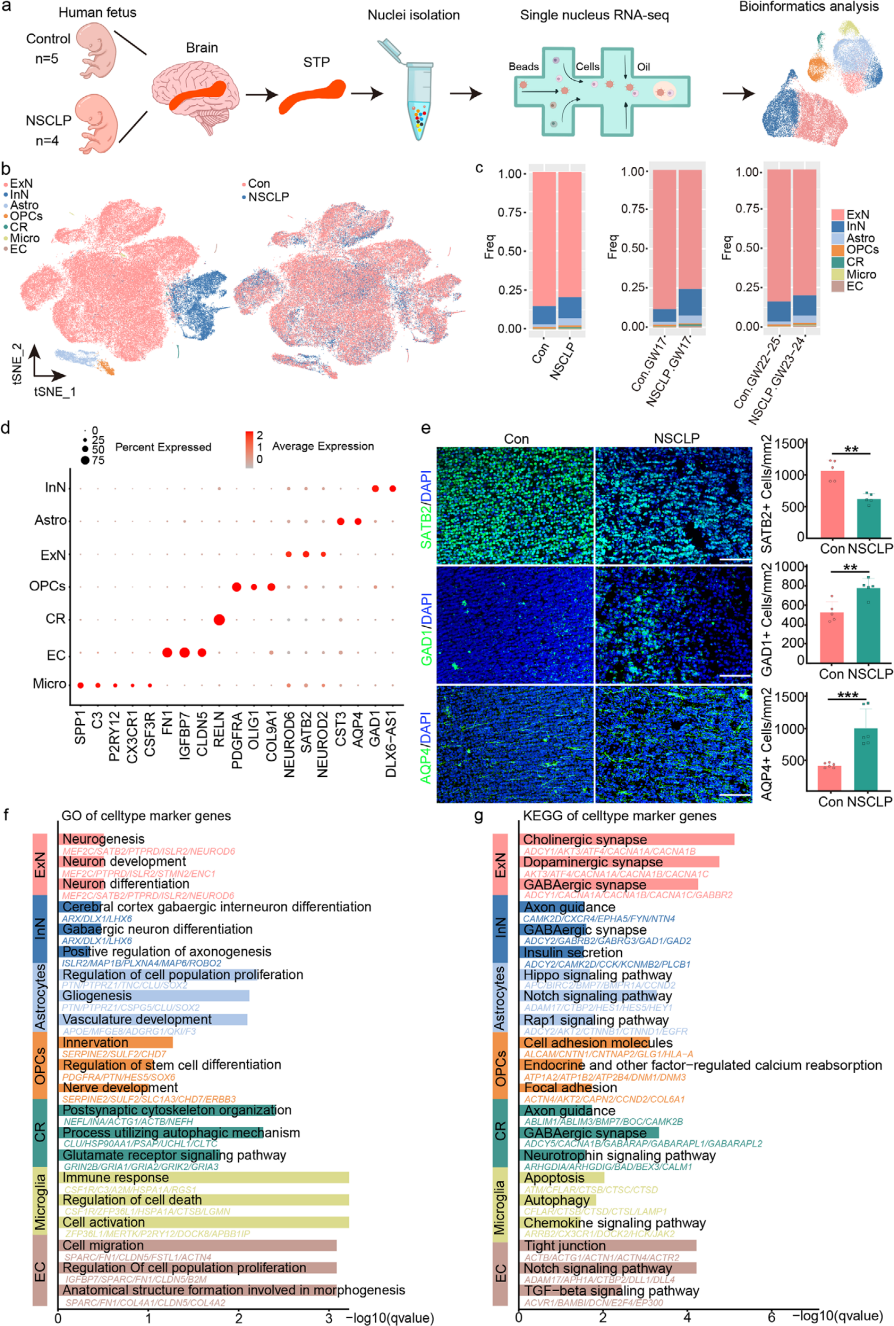
snRNA‐seq reveals cellular heterogeneity in the STP regions of NSCLP fetal brains. a) Schematic workflow of the study, from fetal brain tissue collection to snRNA‐seq analysis. b) t‐SNE plots of single‐cell clustering showing cellular heterogeneity in control (Con, *n* = 5 fetuses) and NSCLP (*n* = 4 fetuses) groups. c) Stacked bar charts showing cell type proportions in each sample (left) and overall comparison between groups (right). d) Dot plot showing the expression of canonical marker genes for cell type annotation. e) Representative immunofluorescence staining of SATB2⁺ ExN, GAD1⁺ InN, and AQP4⁺ Astro in the STP, with quantification of cell density (cells/mm^2^, mean ± SD; Con, *n* = 5–6 sections from 2 fetuses; NSCLP, *n* = 5–6 sections from 2 fetuses). Scale bars, 100 µm. Unpaired two‐sided Student's *t*‐test was applied. ^**^ *p* < 0.01, ^***^ *p* < 0.001. f) GO enrichment analysis of marker genes for each cell type. g) KEGG enrichment analysis of marker genes showing key pathways related to NSCLP neurodevelopment.

To dissect the cellular landscape and transcriptional alterations associated with NSCLP, the STP cortex of fetal brains with NSCLP (*n* = 4 fetuses) and matched controls (Con, *n* = 5 fetuses) was analyzed by snRNA‐seq throughout this study (**Figure** **1**a; Table S1, Supporting Information). In total, 179577 nuclei passed quality control and doublet filtering (67593 from NSCLP and 111984 control nuclei) (Figure 1a). To characterize NSCLP‐related differences in the STP, we first examined whether the composition of the cortex was altered in fetuses with NSCLP. Analysis of normalized cell density by t‐distributed Stochastic Neighbor Embedding (t‐SNE) visualized 15 distinct cell clusters and then integrated into 7 major cell types: excitatory neurons (ExN), inhibitory neurons (InN), astrocytes (Astro), microglia (Micro), oligodendrocyte progenitor cells (OPCs), endothelial cells (ECs), and Cajal‐Retzius (CR) cells (Figure 1b; Figure S1a,b and Table S2, Supporting Information), based on classic maker genes from published literature.^[^ ^19^, ^20^ ^]^ Direct comparison of cell proportions showed a decrease in the proportion of ExN in the NSCLP group (either at gestaional week (GW) 17 or at GW23‐24) when compared to the corresponding stratified Con groups, in particular affecting subtypes belonging to NEUROD6, SATB6 and NEUROD2 (Figure 1c,d; Figure S1c–e, Supporting Information). This was countered by an increase in the fraction of InN belonging to GAD1 and DLX6‐AS1 at GW17, along with Astro augmentation in NSCLP samples relative to Con samples (Figure 1c,d). Immunofluorescence staining consistently validated that, compared with the Con group, the number of SATB2^+^ ExN was significantly decreased, while that of GAD1^+^ InN and AQP4^+^ Astro was notably increased in the NSCLP fetal brain tissues (Figure 1e). This analysis confirmed that NSCLP might be linked to a general decrease in most subtypes of ExN and an increase in InN and Astro. To explore pathways that might underlie NSCLP‐related changes in neuronal function, we selected top up/down‐regulated genes for each cell type and calculated enrichment for Gene Ontology (GO) terms and Kyoto Encyclopedia of Genes and Genomes (KEGG). The most significant GO terms down‐regulated within ExN were related to “neurogenesis,” “neuronal development,” and “neuron differentiation,” whereas down‐regulated genes in InN were involved in “GABAergic interneuron differentiation,” and “axonogenesis.” Besides, strong enrichment of Astro‐associated genes was observed with the biological processes like “regulation of cell population proliferation,” “gliogenesis,” and “vasculature development” (Figure 1f,g; Table S3, Supporting Information).

### 2.2 Global Alteration of Cell–Cell Communication in NSCLP Fetal Brain

**Figure 2:**
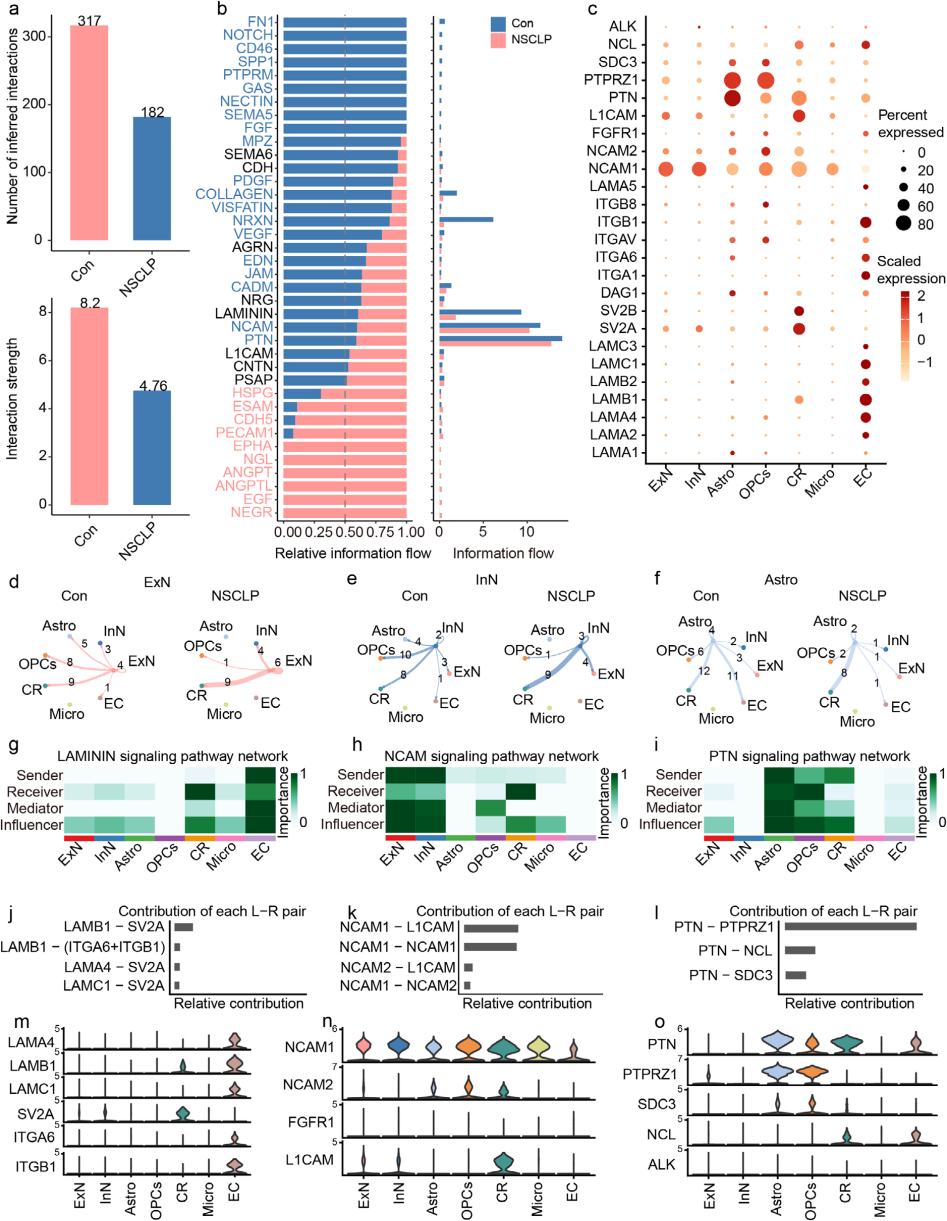
Altered cell–cell communication patterns and signaling pathways in the STP region of fetuses with NSCLP. a) Bar plots showing the total number and overall strength of cell–cell communication interactions in the Con and NSCLP groups. b) Differential activity of major signaling pathways, including LAMININ, NCAM, and PTN, between Con and NSCLP groups. c) Dot plot illustrating the expression patterns of signaling components from the three pathways across seven major cell clusters. d–f) Network diagrams depicting changes in intercellular communication for ExN, InN, and Astro between Con and NSCLP groups. (g–i) Pathway‐level connectivity for LAMININ, NCAM, and PTN pathways, respectively. j–l) Contributions of ligand–receptor (L–R) pairs to the signaling networks. m–o) Violin plots showing cell‐type–specific expression of key L–R pairs in each pathway.

To further explore the cellular interactions in the STP brain region of fetuses with NSCLP, we analyzed cell‐cell communication patterns between Con and NSCLP groups. Cell–cell communication analysis revealed a global reduction in both the number and intensity of cell‐cell interactions in fetuses with NSCLP compared to Con group (**Figure** **2**a). Examination of signaling pathway activity demonstrated selective alterations in information flow, with several pathways associated with extracellular matrix organization, cell adhesion, and neural development enriched in controls, such as FN1‐, NOTCH‐, SPP1, FGF, L1CAM, PTPRM, GAS, NECTIN, and SEMA5, whereas angiogenic and growth factor signaling, including ANGPT, ANGPTL, ESAM, and EGF, were elevated in NSCLP (Figure 2b). Several neurodevelopment‐ and adhesion‐related pathways, including LAMININ (nerve growth and repair), NCAM (neuronal development and migration), NRXN, PTN (neural stem cell regulation), and VEGF, were notably weakened in NSCLP (Figure 2b), suggesting impaired intercellular interactions during craniofacial and neural development. Dot plot visualization of ligand–receptor (L‐P) pair expression highlighted cell‐type–specific contributions to these altered signaling dynamics. NCAM1 was highly expressed in ExN and InN, while Astro expressed PTPRZ1 and PTN, and CR expressed SV2A and SV2B, indicating disrupted extracellular matrix remodeling and neuron–glia adhesion in NSCLP (Figure 2c; Figure S2a–c, Supporting Information).

Network analysis revealed profound rewiring of intercellular signaling in the NSCLP brain microenvironment, with reduced connectivity of ExN, InN and Astro to other cell types in the NSCLP group compared to the Con group, but increased signaling toward CR (Figure 2d–f). Pathway‐specific analysis highlighted a strong enrichment of Laminin signaling in EC, with LAMB1–SV2A L–R pair showing the greatest contribution (Figure 2g,h). NCAM signaling was similarly upregulated in ExN and InN, particularly through the NCAM1–L1CAM and NCAM1‐NCAM1 pairs, implicating enhanced neuronal adhesion and neuron–glia communication (Figure 2j,k). Additionally, PTN signaling emerged as a dominant pathway in Astro, driven by PTN–PTPRZ1 interactions, suggesting a pivotal role in shaping NSCLP‐associated neuronal microenvironment (Figure 2m,n). Notably, PTN–PTPRZ1 communication intensity between Astro–Astro and Astro–OPCs was diminished in NSCLP compared to Con, indicating weakened glial signaling networks (Figure S2d, Supporting Information). Violin plots confirmed cell‐type–specific expression patterns of these pathways (Figure 2i,l,o). Collectively, these findings indicate that ExN, InN and Astro serve as signaling hubs in NSCLP, orchestrating microenvironmental remodeling through Laminin, NCAM, and PTN pathways.

### 2.3 Characterization of ExN Subtypes in NSCLP

**Figure 3:**
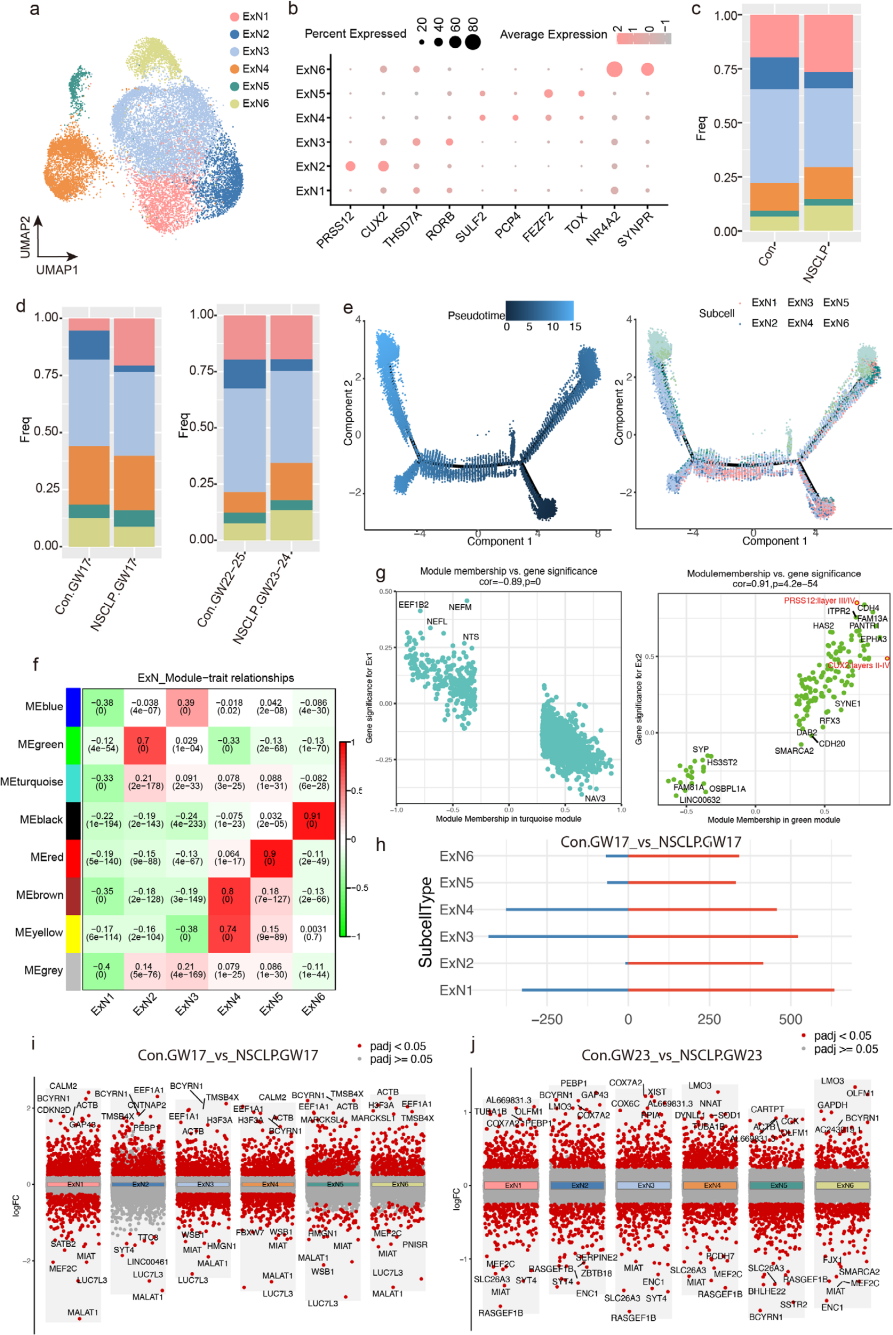
Subtype–specific alterations of ExN in fetuses with NSCLP. a) UMAP plot of ExN subtypes identified in Con (*n* = 5 fetuses) and NSCLP fetuses (*n* = 4 fetuses). b) Dot plot of marker genes used for ExN subtype annotation. c) Bar chart showing the relative proportion of ExN subtypes in Con and NSCLP groups. d) Comparison of ExN subtype composition stratified by gestational weeks (Con.GW17 vs NSCLP.GW17; Con.GW22–25 vs NSCLP.GW23–24). e) Pseudotime trajectory analysis showing ExN differentiation across subtypes. f) WGCNA analysis of module–trait relationships to ExN subtypes. g) Correlation of module membership with gene significance for each ExN subtype. h) Number of dysregulated genes in ExN subtypes between Con.GW17 and NSCLP.GW17. i,j) Volcano plots showing DEGs at GW17 and GW23 between Con and NSCLP groups. Differential expression analyses were performed using model‐based analysis of single‐cell transcriptomics (MAST) with Benjamini–Hochberg correction for multiple testing.

To further dissect the neuronal heterogeneity in fetuses with NSCLP, we performed subcluster analysis on ExN and InN based on snRNA‐seq data. Dimensionality reduction and unsupervised clustering of ExN by Uniform Manifold Approximation and Projection (UMAP) delineated six subtypes (**Figure** **3**a). Marker gene expression patterns indicate laminar assignment of ExN subtypes according to established laminar markers (Figure 3b). CUX2 and RORβ (RORC), enriched in ExN2–ExN3, are canonical markers of upper cortical layers (L2–L4), whereas FEZF2, TOX, and NR4A2, highly expressed in ExN5–ExN6, denote deep‐layer corticofugal neurons (L5–L6).^[^ ^21^, ^22^ ^]^ Notably, SYNPR expression in ExN6 further supports its assignment to layer six corticothalamic neurons.^[^ ^23^ ^]^ Comparison of subtype proportions between NSCLP and Con groups exhibited ExN1, ExN4, ExN5, and ExN6 were increased, while ExN2 and ExN3 showed decreased cell proportions in the NSCLP samples (Figure 3c,d; Figure S3a, Supporting Information), suggesting a potential developmental imbalance between upper‐ and deep‐layer ExN. This laminar organization is consistent with the pseudotime distribution of ExN subtypes (Figure 3e). Subsequent weighted gene co‐expression network analysis (WGCNA) identified gene modules associated with different subtypes: MEgreen showed a positive correlation with ExN2, MEred with ExN5, MEyellow and MEbrown with ExN4, MEblack with ExN6, whereas MEblue was negatively correlated with ExN1 (Figure 3f; Figure S3b,c and Table S4, Supporting Information). Module membership and gene significance analyses highlighted representative hub genes for each module (Figure 3g; Figure S3d–g, Supporting Information). In the MEturquoise module, EEF1B2, NEFL, NEFM, NTS, and NAV3 were strongly and negatively associated with ExN1. Genes such as PRSS12 (L3/4), CUX2 (L2‐4), CDH4, EPHA3, SYNE1, FAM13A, HAS2, ITPR2, PANTR1, RFX3, DAR2, SMARCA2, SYP, HS3ST2, FAM81A, and OSBPL1A within the Megreen module were positively associated with ExN2. The MEblue module was enriched for CSMD2, MAGI2, KCNH7, RORB (L4/5), THSD7A (L2‐4), NYAP2, PDE4B, PCYOX1, and RASGEF1B, showing strong positive correlation with ExN3. The MEbrown module genes, including TLE4, SYT6, GRIK3, TRPM3, ARID1B, NPY, MET, and PENK, were associated with ExN4. Finally, genes in the MEblack module, such as B2M, SYNPR (layer VI), NR4A2 (layer VI), and HTR2C, were positively correlated with ExN6. These findings delineate subtype–specific gene co‐expression programs aligned with known cortical layer markers, underscoring transcriptional specialization across ExN subpopulations. Analysis of differentially expressed genes (DEGs) across ExN subtypes revealed marked temporal differences between NSCLP and Con. Across developmental stages, ExN1, ExN3, and ExN4 consistently exhibited a high burden of downregulated DEGs in NSCLP compared with Con at both GW17 and GW23, indicating persistent transcriptional repression in these ExN subtypes (Figure 3h–j; Figure S3h, Supporting Information). Functional annotation of these downregulated genes revealed subtype–specific vulnerabilities: ExN1 genes were enriched for pathways related to synaptic vesicle organization and neurotransmitter release, suggesting impaired synaptic transmission; ExN3 downregulated genes were associated with cerebral cell migration and glutamatergic signaling, implicating disrupted neuronal positioning and excitatory circuit formation; and ExN4 showed repression of genes linked to GABA receptor activity, pointing to potential deficits in inhibitory synaptic function (Figure S3i–n, Supporting Information). Together, these results highlight sustained and subtype–specific transcriptional dysregulation in NSCLP, with ExN subtypes displaying distinct molecular signatures that may converge on disrupted excitatory‐inhibitory balance during cortical development.

### 2.4 Feature of InN Subtypes in NSCLP

**Figure 4:**
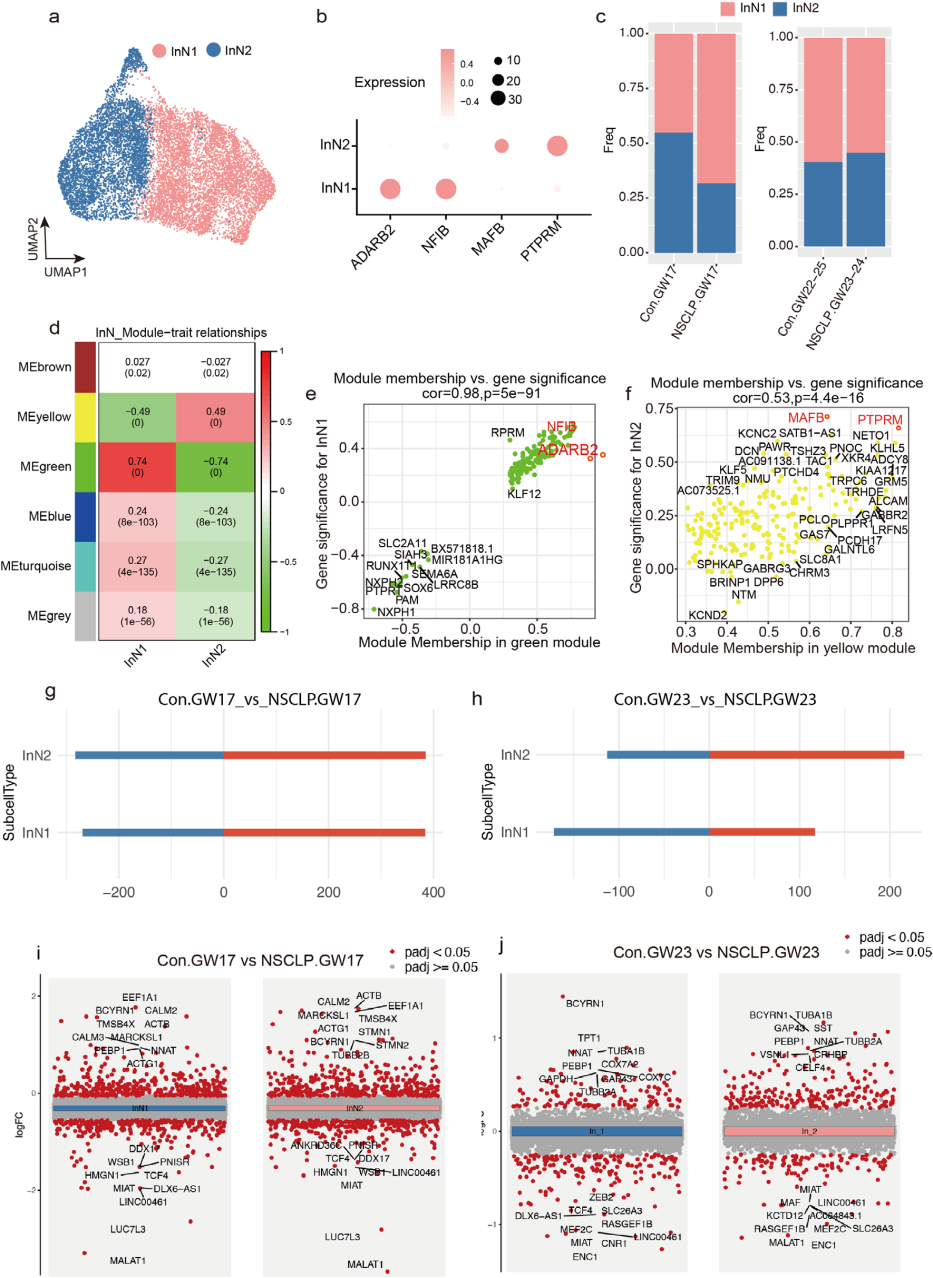
Subtype–specific alterations of InN and dysregulated transcriptional programs in fetuses with NSCLP. a) UMAP plot of InN subtypes in Con (*n* = 5) and NSCLP fetuses (*n* = 4). b) Dot plot showing marker gene expression used to define InN subtypes. c) Bar chart comparing InN subtype proportions stratified by gestational weeks (Con.GW17 versus NSCLP.GW17; Con.GW22–25 vs NSCLP.GW23–24). d) WGCNA analysis of module–trait relationships to InN subtypes. e,f) Correlation between module membership and gene significance for InN subtypes. g,h) Numbers of dysregulated genes in InN subtypes between Con.GW17 and NSCLP.GW17, and between Con.GW23 and NSCLP.GW23. i,j) Volcano plots showing dysregulated genes in InN subtypes at GW17 and GW23. DEGs were identified using MAST with adjusted *p* < 0.05 (Benjamini–Hochberg correction).

For InN, clustering revealed two transcriptionally distinct subtypes (InN1 and InN2; **Figure** **4**a,b). InN1, characterized by high ADARB (RNA editing enzyme) and NFIB (a transcription factor critical for gliogenesis and forebrain development) expression, likely represents a subtype enriched in transcriptional regulators and RNA metabolism, suggestive of neurons with higher transcriptional plasticity or developmental potential. In contrast, InN2, defined by elevated MAFB (involved in interneuron specification and differentiation) and PTPRM (a cell adhesion molecule implicated in synaptic organization), may correspond to a more mature or synaptically specialized interneuron population (Figure 4b). Notably, InN1, accounting for larger proportions during development than InN1, increased in the NSCLP group at GW17, whereas those of InN2 increased in the NSCLP samples compared with Con at GW22‐25 (Figure 4c; Figure S4a, Supporting Information), suggesting a possible shift in InN subtype composition associated with disease‐related cortical development alterations. WGCNA further identified MEyellow as the module most strongly correlated with InN1 and MEgreen with InN2 (Figure 4d; Table S4, Supporting Information). Correlation of module eigengenes with subtype marker genes confirmed a strong association: ADARB and NFIB with InN1, and MAFB and PTPRM with InN2 (Figure 4e,f; Figure S4b,c, Supporting Information), validating our subtype annotations and reflecting established interneuron subtype markers.^[^ ^24^, ^25^ ^]^ Compared with GW17, the number of DEGs between NSCLP and Con samples in InN1 and InN2 markedly decreased at GW23 (Figure 4g–j). At GW17, DEGs in both subtypes were predominantly enriched in synaptic signaling pathways, including GABAergic, glutamatergic, cholinergic, serotonergic, and dopaminergic transmission, suggesting widespread perturbations in neurotransmitter systems at early developmental stages (Figure S4d, Supporting Information). By contrast, at GW23, InN1 DEGs were primarily associated with neuronal development and differentiation, whereas InN2 DEGs were enriched for pathways related to neuronal apoptosis and neurodegenerative disorders, indicating a shift from early synaptic dysfunction to later‐stage neuronal vulnerability in NSCLP (Figure S4e, Supporting Information).

### 2.5 Hub Transcription Factors and Regulatory Networks Underlying NSCLP‐Associated Alterations in ExN

**Figure 5:**
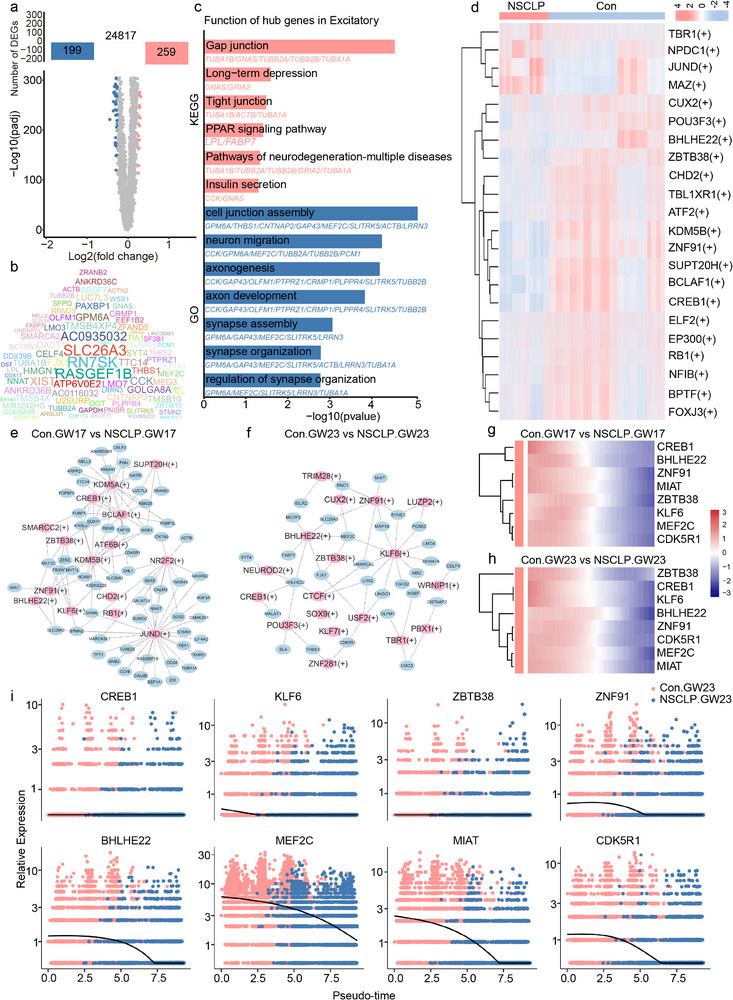
Identification of hub TFs and regulatory networks underlying ExN dysfunction in NSCLP. a) Volcano plot of DEGs in ExN between Con (*n* = 5) and NSCLP fetuses (*n* = 4). b) Machine learning–derived hub gene cloud map showing NSCLP‐associated ExN genes, scaled by feature importance. c) GO and KEGG enrichment analysis of NSCLP‐related hub genes (adjusted *p* < 0.05, Benjamini–Hochberg correction). d) Heatmap of key TFs in ExN identified by SCENIC analysis. e,f) Transcriptional regulatory networks of TFs and target genes in ExN at GW17 and GW23 in Con and NSCLP groups. g,h) Heatmap showing the expression patterns of hub TFs and their targets in ExN at GW17 and GW23. i) Pseudotime trajectory analysis displaying dynamic expression of CREB1, BHLHE22, ZNF91, ZBTB38, KLF6, MIAT, MEF2C, and CDK5R1 during ExN maturation. Pseudotime trajectories were inferred using Monocle 2. Statistical analysis was performed using MAST for DEGs, with Benjamini‐Hochberg adjusted *p* values reported.

To elucidate transcriptional regulators underlying neurodevelopmental abnormalities in the STP of fetuses with NSCLP, we integrated snRNA‐seq analysis with machine learning–based hub gene prioritization and SCENIC regulatory network inference (Figure S5, Supporting Information). Differential expression analysis of ExN cells identified 259 upregulated and 199 downregulated genes in NSCLP compared with Con (**Figure** **5**a). Machine learning approaches highlighted a hub gene set enriched for pathways related to axonogenesis, synapse assembly, structural regulation, gap junctions, apoptosis, and neurodegeneration (Figure 5b,c). SCENIC analysis displayed different patterns of transcription factor (TF)‐target interactions between the NSCLP group and Con group, which revealed TFs including CREB1, ZBTB38, BHLHE22, KLF6, and ZNF91, consistently detected across both GW17 and GW23 in Con and NSCLP samples (Figure 5d–f). Integration of pseudotime analysis demonstrated significant downregulation of these TFs and their targets, such as MEF2C, MIAT, and CDK5R1, in NSCLP, with expression progressively declining along developmental trajectories (Figure 5g–i). Functional enrichment of TF target genes highlighted a shift from developmental programs to synapse‐associated functions as NSCLP progressed, suggesting stage‐specific dysregulation of ExN maturation (Figure S6a–h, Supporting Information). Finally, we analyzed the TF–hub gene interaction networks of the Con and NSCLP groups separately (Figure S6i–l, Supporting Information), revealing stage‐specific transcriptional dynamics. Notably, its downstream target MEF2C remained consistently downregulated in NSCLP, suggesting that dysregulated MEF2C signaling may contribute to disrupted ExN maturation.

### 2.6 Hub TFs and Regulatory Networks Vulnerable to NSCLP in InN

**Figure 6:**
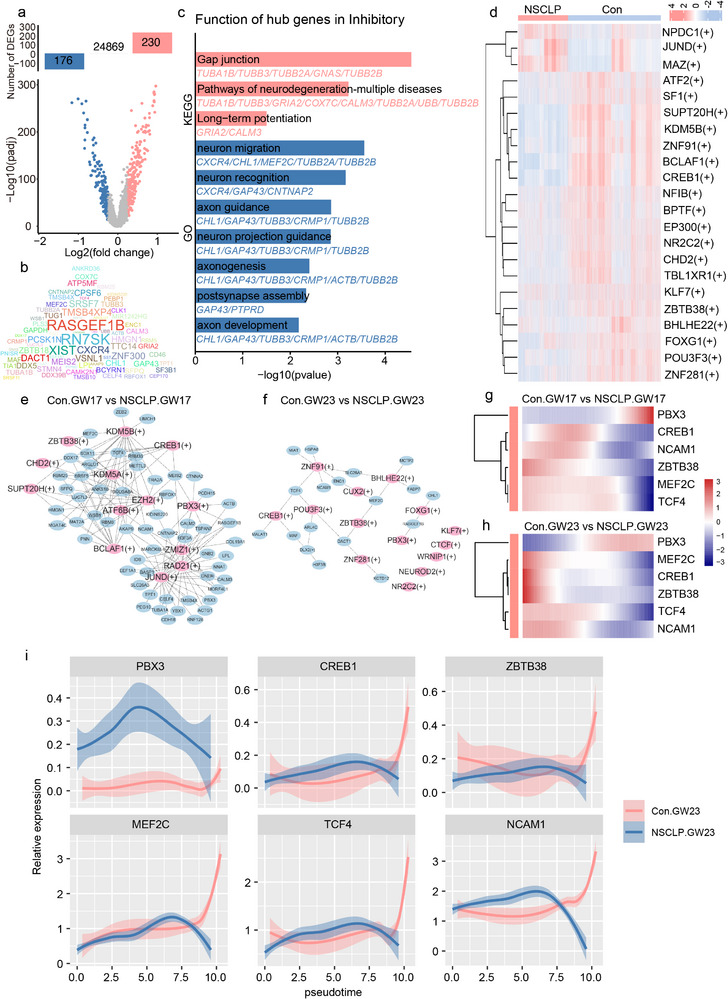
Hub TFs and regulatory networks contributing to InN abnormalities in NSCLP. a) Volcano plot of DEGs in InN Con (*n* = 5) and NSCLP groups (*n* = 4). Red dots represent upregulated genes and blue dots represent downregulated genes (adjusted *p* < 0.05, |log_2_FC| > 0.25, Benjamini–Hochberg correction). b) Machine learning‐derived hub gene cloud map identifying InN‐specific NSCLP‐associated hub genes, sized by feature importance. c) GO and KEGG enrichment analysis of InN‐associated hub genes (Benjamini‐Hochberg adjusted *p* < 0.05). d) Heatmap of key TFs identified in InN via SCENIC analysis at GW17 and GW23. e,f) Regulatory networks of TFs and target genes in InN at GW17 and GW23. g,h) Heatmap showing expression of hub TFs and targets across developmental stages. i) Pseudotime trajectory analysis of InN showing dynamic expression of PBX3, CREB1, ZBTB38, NCAM1, MEF2C, and TCF4. Pseudotime trajectories were inferred using Monocle 2. DEGs were tested using MAST with Benjamini–Hochberg correction.

SnRNA‐seq analysis of InN revealed 230 upregulated and 176 downregulated genes in the NSCLP group compared with Con group (**Figure** **6**a). To further prioritize key regulators, we applied machine learning models (CatBoost) to classify DEGs across developmental stages (GW17 vs. GW23) and between groups (Con vs NSCLP) (Figure S7, Supporting Information). Performance evaluation, learning curves, and feature selection analysis confirmed the robustness of the model and enabled the identification of hub genes most relevant to NSCLP pathology. These hub genes were also identified in both Con and NSCLP fetal STP and visualized using cloud term plots (Figure 6b). Functional enrichment of hub genes highlighted biological processes associated with neuronal recognition, axonal projection guidance, synaptic function, and cognition, with KEGG pathways enriched in Gap junction, neurodegeneration, cAMP signaling, and dopaminergic synapse (Figure 6c). Further enrichment analysis of hub genes from Con (GW17 vs. GW23) and NSCLP (GW17 vs. GW23) groups revealed similar functional signatures, emphasizing roles in synapse formation, neuronal migration, and stem cell differentiation (Figure S8a–h, Supporting Information). To explore upstream transcriptional regulation, SCENIC analysis was performed to construct TF–target gene regulatory networks in InN at both GW17 and GW23 (Figure 6d–f). Hub TFs, including PBX3, CREB1, and ZBTB38, were consistently downregulated in NSCLP and regulated key target genes such as NCAM1, MEF2C, and TCF4 (Figure 6g–i; Figure S8i–l, Supporting Information). Machine learning analysis of Astro did not reveal overlapping hub TFs or targets consistently associated with NSCLP (Figures S9–S11, Supporting Information). Therefore, subsequent analyses focused on ExN and InN as the main contributors to NSCLP‐related neurodevelopmental abnormalities.

### 2.7 MEF2C Downregulation Links Synaptic Dysfunction to Neurodevelopmental Abnormalities

**Figure 7:**
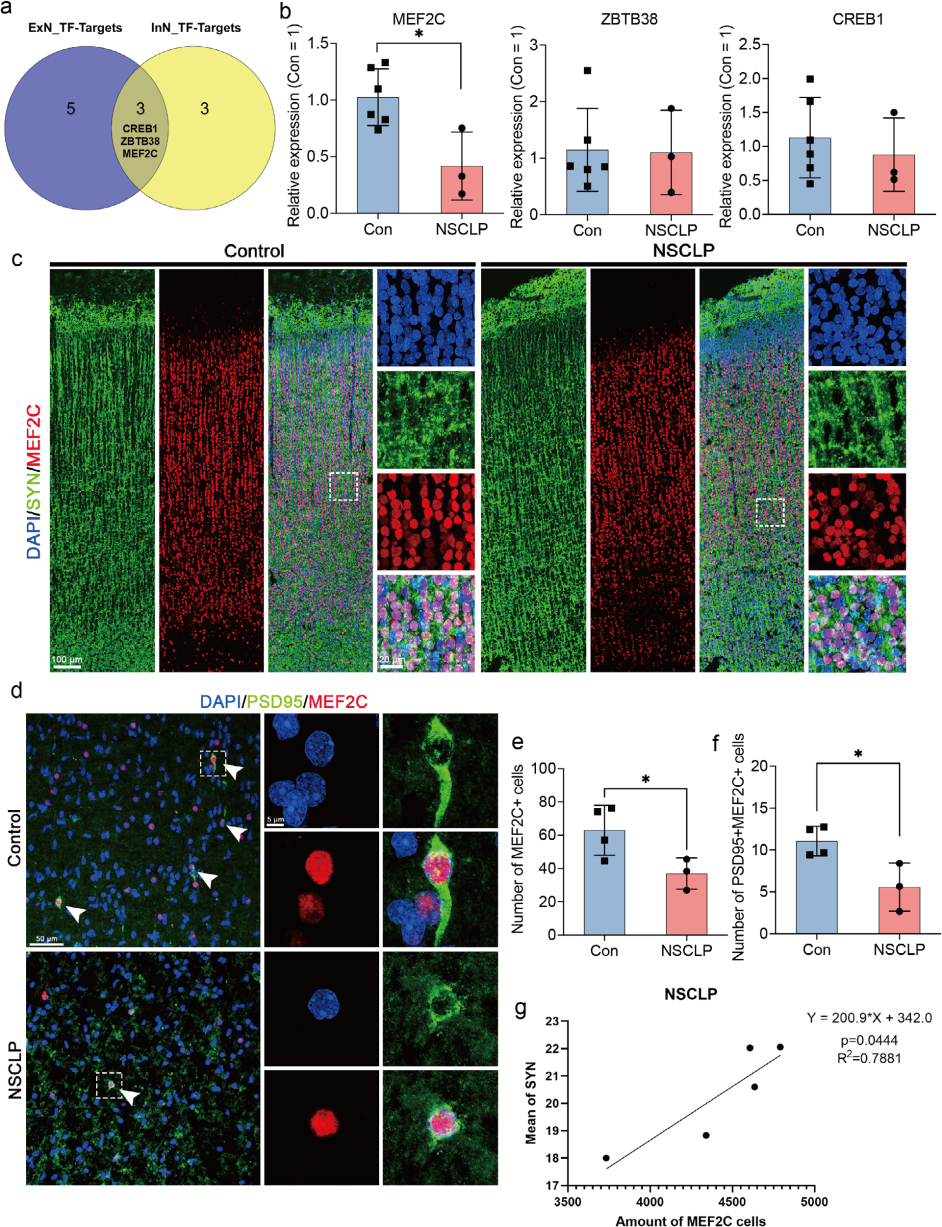
Validation of MEF2C dysregulation and its association with synaptic deficits in NSCLP fetal brain tissues. a) Venn diagram showing the overlap of TFs and target genes between ExN and InN, highlighting MEF2C, CREB1, and ZBTB38 as shared regulators. b) RT‐qPCR validation of key TFs (CREB1, ZBTB38) and target gene (MEF2C) in fetal brain tissues (Con, *n* = 6 fetuses; NSCLP, *n* = 3 fetuses). Each dot represents one biological replicate (four technical replicates averaged). Data are mean ± SD. Unpaired two‐sided Student's *t*‐test with Benjamini–Hochberg correction was applied. MEF2C: *p* = 0.0142; *p_*adj = 0.0427. c,d) Representative immunofluorescence staining of MEF2C with SYN (c) and PSD95 (d) in fetal brain tissues. Dashed boxes indicate magnified regions. Scale bars: left, 100 µm (c) or 50 µm (d); right, 20 µm (c) or 5 µm (d). e,f) Quantification of MEF2C⁺ cells (e) and PSD95⁺MEF2C⁺ cells (f) in Con (*n* = 4 sections from two fetuses, three field views per section) and NSCLP (*n* = 3 sections from two fetuses, three field views per section) fetal brain tissues (f, 95% confidence interval, −9.949 to −1.033). Data are mean ± SD; unpaired two‐sided Student's *t*‐test. ^*^ *p* < 0.05. g) Pearson correlation between MEF2C expression and SYN intensity in NSCLP fetal brain tissues.

To uncover key transcriptional regulators underlying neurodevelopmental abnormalities in the STP region of NSCLP fetuses, we took intersections of TF‐target genes of ExN and InN populations, identifying two key TFs (CREB1 and ZBTB38) and one target gene, MEF2C (**Figure** **7**a). MEF2C has been implicated in neuronal development in previous studies,^[^ ^26^ ^]^ while its role in NSCLP remains unclear. We assessed the expression levels of CREB1, ZBTB38, and MEF2C in NSCLP fetal brain tissue using real‐time quantitative polymerase chains reaction (RT‐qPCR). Despite the reduced expression of CREB1 and ZBTB38 in NSCLP samples compared to Con group, these differences were not statistically significant (Figure 7b). In contrast, MEF2C expression was significantly downregulated in NSCLP fetal brain tissue, consistent with our snRNA‐seq data (Figure 7b). Immunofluorescence staining further confirmed a significant reduction of MEF2C expression in NSCLP samples (Figure 7c,e). Given that MEF2C's enrichment in synaptic structure and function, we continued to investigate its association with synaptic integrity. Double immunofluorescence staining for MEF2C and synaptic markers, including synaptophysin (SYN) and postsynaptic density protein‐95 (PSD95), demonstrated a significant decrease in PSD95 expression in NSCLP fetal brain tissue (Figure 7c–f). Correlation analysis revealed a positive correlation between MEF2C and SYN expression, suggesting that MEF2C downregulation contributes to impaired synaptic structure and function (Figure 7g). Collectively, these findings indicate that reduced MEF2C expression is strongly linked to synaptic dysfunction in NSCLP fetal brain tissue, providing insight into the neurodevelopmental deficits observed in this disorder.

### 2.8 MEF2C Knockdown Impairs Excitatory Synaptic Function and Transmission

**Figure 8:**
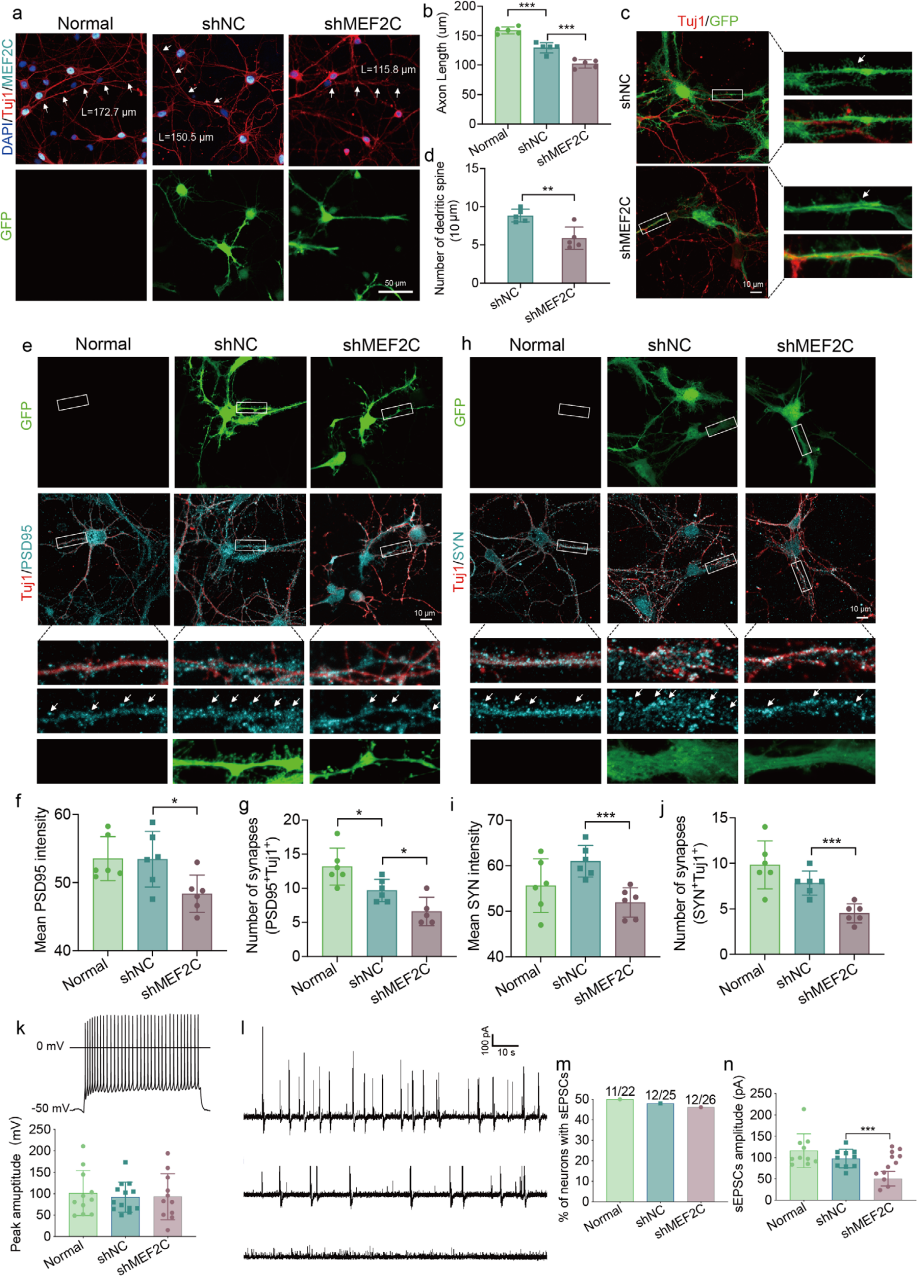
Functional impairment of neuronal and synaptic activity upon MEF2C knockdown in primary cortical neurons. a) Immunostaining of Tuj1/MEF2C in neurons transduced with control shRNA (shNC) or MEF2C‐shRNA (shMEF2C). Scale bar, 50 µm. Green, GFP fluorescence. White arrows indicate axons. b) Quantification of neuronal axon length among Normal, shNC, shMEF2C groups. *n* = 5 coverslips. Data are mean ± SD; one‐way ANOVA with Tukey's post hoc test. c) Representative immunofluorescence images of Tuj1 in shNC and shMEF2C neurons. Green, GFP fluorescence. Scale bar, 10 µm. White arrows indicate dendritic spines. d) Quantification of dendritic spines of neurons in shNC and shMEF2C groups. *n* = 5 coverslips. Data are mean ± SD; Student's *t*‐test. e,h) Representative immunofluorescence images of Tuj1/PSD95 and Tuj1/SYN in Normal, shNC and shMEF2C neurons. Green, GFP fluorescence. Scale bar, 10 µm. White box indicates interest area. f–j) Quantification of synapse amount and mean density in Normal, shNC, shMEF2C groups. *n* = 6 coverslips. Data are mean ± SD; one‐way ANOVA with Tukey's post hoc test. k) Quantification of peak amplitude of action potentials under 190 pA current injection. *n* = 9–13 neurons. Data are mean ± SEM; one‐way ANOVA with Tukey's post hoc test. l) Representative traces of sEPSCs recorded at −60 mV. m) Percentage of neurons exhibiting sEPSCs (Con, *n* = 12 neurons; shNC, *n* = 11 neurons; shMEF2C, *n* = 12 neurons). n) Quantification of sEPSC amplitude (5 min recording per neuron; *n* = 11–12 neurons per group). Data are mean ± SEM; one‐way ANOVA with Tukey's post hoc test. ^*^ *p* < 0.05, ^**^ *p* < 0.01, and ^***^ *p* < 0.001.

To further evaluate synaptic integrity and function, we performed immunostaining for Tuj1, SYN and PSD95 in MEF2C‐deficient neurons. The MEF2C knockdown group exhibited a clear decrease in both axon length and number of dendritic spine of neurons compared to Normal and shNC group (**Figure** **8**a–d), indicating deficits in synaptic formation. Additional evidence demonstrated the reduction of the number of synapses and mean intensity of PSD95 and SYN in MEF2C‐deficient neurons (Figure 8e–j), further highlighting synaptic impairment. To investigate the role of MEF2C in neuronal function, we performed whole‐cell patch‐clamp recordings on primary cortical neurons following lentivirus‐mediated MEF2C knockdown. Analysis of evoked action potentials revealed no significant differences in peak amplitude among the Normal, shNC, and shMEF2C groups (Figure 8k), indicating that MEF2C depletion is less associated with basic electrophysiological properties than with synaptic transmission and network integration. In contrast, recordings of spontaneous excitatory postsynaptic currents (sEPSCs) showed a reduction in the proportion of neurons exhibiting sEPSCs in the shMEF2C group (Figure 8l,m). Moreover, we observed a significant decrease in sEPSC amplitude in MEF2C‐deficient neurons (Figure 8n). These findings indicate that MEF2C is essential for maintaining excitatory synaptic integrity, potentially through coordinated regulation of both presynaptic release machinery and postsynaptic receptor composition.

## 3 Discussion

In the present study, snRNA‐seq was employed to characterize the cellular heterogeneity of STP region in fetuses with NSCLP. Detailed investigation identified seven major cell types, including ExN, InN, Astro, Micro, OPCs, ECs, and CRs. Differential gene expression analysis further dissected disease‐associated transcriptional changes. The integration of single‐nucleus transcriptomics and machine learning algorithms allowed the construction of TF–target networks and identified MEF2C as a key regulator of neuronal development. Notably, progressive downregulation of MEF2C in NSCLP samples was found to positively correlate with synaptic function, potentially contributing to neurodevelopmental abnormalities. In vitro functional assays demonstrated markedly impaired synaptic integrity, indicated by reduced levels of SYN and PSD95. Electrophysiological recordings further demonstrated a reduction in the proportion of neurons exhibiting sEPSCs and a pronounced decrease in sEPSC amplitude, suggesting that MEF2C regulates presynaptic protein function and directly or indirectly modulates the expression of postsynaptic genes to maintain synaptic transmission. Collectively, these findings suggest that MEF2C downregulation might contribute to synaptic dysfunction and neurodevelopmental abnormalities in NSCLP. This study highlights that, beyond overt craniofacial malformations, NSCLP may also involve underlying brain dysfunction, underscoring the need for deeper investigation into its molecular mechanisms.

Previous studies have provided compelling evidence that NSCLP is not only a craniofacial malformation but may also be associated with neurodevelopmental abnormalities. A study in 2018 reported marked structural abnormalities in the brains of both human patients and mice with orofacial clefts of the lip and/or palate, along with seriously impaired development of GABAergic cortical interneurons.^[^ ^27^ ^]^ This finding lends further credence to the hypothesis that NSCLP may be associated with neurodevelopmental defects. Additionally, mutations in the PAH gene, which is implicated in disorders affecting neurodevelopment, further support the potential link between NSCLP and neurological deficits.^[^ ^28^ ^]^ In line with these studies, our findings indicated the presence of neurodevelopmental abnormalities in the STP brain region of the fetuses in the NSCLP group, characterized by altered proportions of ExN and InN, dysregulation of neurodevelopmental pathways, and differential expression of genes related to synaptic development and cortical patterning. These results provide direct molecular evidence of disrupted brain development in NSCLP. Cognitive deficits in patients with CLP have historically been underestimated, but rowing evidence shows that patients with NSCLP have lower Full Scale Intelligence Quotient (FSIQ) scores compared to healthy controls^[^ ^29^ ^]^ and are more prone to neurodevelopmental and academic difficulties.^[^ ^30^ ^]^ Structural neuroimaging studies have provided direct evidence of poor brain development in patients with NSCLP,^[^ ^31^, ^32^ ^]^ including abnormalities in the ventral medial prefrontal cortex (vmPFC) in male patients with CLP, which may contribute to their social and cognitive impairments.^[^ ^31^ ^]^ Moreover, Shriver et al. reported that increased STP volume of in adult male patients with CLP was associated with decreased IQ, supporting the link between cognitive dysfunction and craniofacial malformation.^[^ ^33^ ^]^ These findings, together with ours, reinforce the potential presence of neurodevelopmental defects in NSCLC and suggest that such neurodevelopmental abnormalities may underlie the observed cognitive dysfunction.

In addition to transcriptional dysregulation, our analysis uncovered profound alterations in cell–cell communication within the STP microenvironment of fetuses with NSCLP, further underscoring disrupted brain development. We observed a global attenuation of intercellular communication in NSCLP, with marked reductions in both the number and strength of ligand–receptor interactions compared with controls. Pathways linked to extracellular matrix (ECM) organization and axon guidance—including Laminin, NCAM, and PTN signaling—were notably weakened, while angiogenic pathways such as ANGPT and growth factor signaling were upregulated, suggesting a compensatory remodeling response. The attenuation of Laminin and NCAM pathways is particularly relevant, as Laminin family proteins are essential for axon guidance, neuronal migration, and synaptic plasticity, and their disruption has been implicated in neurodevelopmental disorders and craniofacial malformations. Similarly, NCAM‐mediated adhesion is critical for neuronal differentiation and circuit formation, with altered NCAM expression reported in disorders such as autism spectrum disorder and intellectual disability. PTN–PTPRZ1 signaling, which regulates neural stem cell maintenance and astrocytic interactions, was also markedly weakened, consistent with impaired neuron–glia communication during cortical development. The increased angiogenic signaling observed in NSCLP may reflect a microenvironmental compensation for impaired neuronal connectivity, as vascular remodeling has been linked to neurodevelopmental defects in both human and animal models.^[^ ^34^ ^]^ Network analysis further identified ExN, InN, and Astro as key communication hubs, orchestrating the developmental microenvironment. The reduced connectivity of ExN and InN aligns with previous observations of altered excitatory/inhibitory ratios, reinforcing the hypothesis that disrupted neuronal network organization underlies cognitive and neurodevelopmental phenotypes in NSCLP. These findings parallel previous reports of neuronal migration deficits and disrupted cortical layering in CLP animal models,^[^ ^27^ ^]^ and extend them by mapping the signaling rewiring that accompanies structural abnormalities. Collectively, these results integrate transcriptomic and communication‐level evidence to support a model in which NSCLP is associated with impaired ECM remodeling, defective neuron–glia signaling, and angiogenic compensation, ultimately contributing to abnormal craniofacial and cortical development. By combining single‐nucleus transcriptomics with intercellular network analysis, this study provides a molecular framework linking craniofacial malformations to cognitive deficits and neurodevelopmental vulnerabilities reported in NSCLP patients.

Function enrichment analysis of hub genes in ExN revealed a strong enrichment of pathways related to synapse formation, synaptic organization and structural regulation, supporting the notion that synaptic abnormalities may be a crucial feature of neurodevelopmental impairment in fetuses with NSCLP. This is consistent with existing studies reporting that genes associated with CLPs, such as CLPTM1, may interact with GABAergic receptors and modulate synaptic levels.^[^ ^35^, ^36^ ^]^ To dissect the regulatory mechanisms underlying neurodevelopmental abnormalities in NSCLP, we focused on TFs and their downstream target genes that were persistently downregulated across both ExN and InN neuronal populations at multiple developmental stages. The STP region is a critical site of coordinated excitatory‐inhibitory balance during neurodevelopment.^[^ ^37^, ^38^ ^]^ Dysregulation of transcriptional programs shared across these two neuronal lineages may therefore represent convergent mechanisms underlying NSCLP‐associated abnormalities. By examining shared regulatory disruptions across two neuronal subtypes, we aimed to identify core transcriptional programs contributing to global neurodevelopmental vulnerability rather than cell‐type–specific effects. Moreover, TFs with broad influence across neuronal subtypes may exert amplified effects on network assembly, synaptic organization, and circuit function, all of which are potentially relevant to the etiology of NSCLP. In our study, MEF2C emerged as a key regulatory node, showing robust and persistent downregulation in both neuronal lineages. While this intersectional approach effectively highlights core regulators, we acknowledge that lineage‐specific TFs crucial for subtype–specific maturation or circuit integration may have been underrepresented. Future investigations employing cell‐type–specific perturbation models and higher‐resolution single‐cell regulatory network inference could help uncover such lineage‐restricted regulators and refine our understanding of cell‐type–specific contributions to NSCLP pathogenesis.

MEF2C haploinsufficiency has been associated with severe intellectual disability and autism spectrum disorder, where MEF2C loss disrupts synaptogenesis, glutamatergic signaling, and microglial activity.^[^ ^26^, ^39^, ^40^ ^]^ MEF2C activation is triggered by calcium influx following neurotrophin stimulation and synaptic activity, which in turn activates gene expression programs that modulate synaptic plasticity.^[^ ^41^ ^]^ During the early stages of neurodevelopment, MEF2C regulates the differentiation of specific neuronal subtypes and the expression of synaptic genes.^[^ ^42^ ^]^ Common genetic variants in MEF2C are also implicated in schizophrenia, cognitive traits, autism spectrum disorders, epilepsies, and Alzheimer's disease,^[^ ^26^, ^41^ ^]^ underscoring its broad involvement in neuropsychiatric disorders. In this study, we combined snRNA‐seq analysis, machine learning prioritization, and fetal brain validation to confirm MEF2C as an important factor in NSCLP neuropathology. Importantly, functional validation in primary cortical neurons demonstrated that MEF2C knockdown significantly impairs synaptic formation and neuronal function, further supporting its possible role in neurodevelopmental processes related to NSCLP. Moreover, MEF2C depression significantly reduced the amplitude of sEPSCs, indicating impairments in both presynaptic and postsynaptic function. These results provide experimental evidence that MEF2C dysregulation could possibly compromise synaptic assembly and neuronal network function in NSCLP.

Nevertheless, the functional validation was performed primarily in primary neuronal cultures rather than in disease‐specific models of NSCLP. This approach was necessitated by the fact that currently available in vivo and in vitro models for human NSCLP remain imperfect in recapitulating disease pathogenesis and are not yet widely adopted for systematic mechanistic investigation. In response to this challenge, our team is currently developing human‐derived craniofacial cleft organoids, which we anticipate will provide a more robust and clinically relevant platform for future validation and deeper mechanistic exploration. Despite these limitations, our findings strongly support the role of MEF2C dysregulation in synaptic abnormalities observed in human NSCLP tissues. Future work will focus on leveraging these emerging human‐relevant model systems to further elucidate the pathogenic cascades underlying NSCLP.

Upstream regulatory analysis further positioned MEF2C within a conserved neurodevelopmental transcriptional hierarchy. We have analyzed transcriptional regulatory networks and identified several TFs that are predicted to act upstream of MEF2C. Specifically, KLF6, ZBTB38, and BHLHE22 were identified in ExN at both GW17 and GW23, while CUX2, BHLHE22, and ZBTB38 regulated MEF2C in both ExN and InN at GW23. KLF6 is known to regulate neuronal differentiation and axonal regeneration,^[^ ^43^ ^]^ suggesting it may contribute to initiating MEF2C expression during cortical development. BHLHE22 plays a role in controlling a program of gene expression that mediates aspects of neural development, including axonal outgrowth,^[^ ^44^ ^]^ which may influence MEF2C activity in establishing excitatory‐inhibitory balance. CUX2 is critical for neuronal migration and dendritic branching, processes that overlap with MEF2C‐mediated synaptic regulation.^[^ ^45^ ^]^ ZBTB38 is involved in DNA methylation‐dependent transcriptional regulation and has been linked to cell survival and neurogenesis,^[^ ^46^ ^]^ raising the possibility that epigenetic mechanisms contribute to MEF2C regulation. Notably, ZBTB38 appeared as a consistent upstream regulator in both ExN and InN across multiple developmental stages, suggesting a potentially stable and epigenetically mediated regulatory axis. These results indicate that MEF2C dysregulation may be orchestrated by multilayered regulatory networks integrating transcriptional and epigenetic cues. Although several upstream transcriptional regulators of MEF2C were inferred from network analyses, the downstream regulatory network of MEF2C—particularly its target genes and functional effects—remains uncharacterized in this study. Future studies employing cell‐type–specific perturbation models, CRISPR‐based regulatory screening, and integrative single‐cell regulatory network inference will be essential to e experimentally validate upstream regulators and map the full scope of MEF2C's upstream and downstream regulation in NSCLP pathogenesis.

## 4 Conclusion

In this study, we leveraged snRNA‐seq data from NSCLP fetal brain tissue to construct a single‐cell transcriptomic landscape of the STP region during mid‐gestation, uncovering cell‐type–specific transcriptional changes underlying NSCLP progression. By employing WGCNA and SCENIC analyses, we identified disease‐associated gene modules and transcriptional regulatory networks, ultimately pinpointing MEF2C as a critical transcription factor whose downregulation significantly disrupts synaptic function and neuronal development. The reduced expression of MEF2C were further validated in NSCLP fetal brain samples, and subsequent in vitro functional assays further established its essential role in neurodevelopment. Together, these findings provide new mechanistic insights into the interplay between craniofacial and brain development, laying a foundation for future diagnostic and therapeutic strategies aimed at mitigating cognitive deficits associated with this disorder.

## 5 Experimental Section

### Human Fetus Brain Specimen Collection

All human subject‐related research procedures in this study were approved by the Clinical Trial and Biomedical Ethics Committee of the Affiliated Hospital of Zunyi Medical University (approval No.: KLL‐2022–446) with Chinese Clinical Trial Registry (No. ChiCTR2300070041, Date: 03/31/2023, Prospective registration, https://www.chictr.org.cn/showproj.html?proj = 193 997). This study was conducted in line with the guidelines of the Declaration of Helsinki. A total of 5 Con fetuses (2 at GW17, 1 at GW22, 1 at GW23 and 1 at GW25) and 4 fetuses with NSCLP (1 at GW17, 2 at GW23 and 1 at GW24) were enrolled in the snRNA‐seq experiment. For NSCLP samples, when the fetuses were prenatally diagnosed with NSCLP by experienced clinicians using color Doppler ultrasound, clinical recommendations for elective termination are generally made typically before GW25 with parental informed consent. Con fetuses were selected from elective terminations of pregnancies that did not have any known congenital abnormalities, including cleft lip or palate. All fetuses, including both NSCLP and Con groups, were obtained from elective terminations at comparable gestational ages. To control potential confounding variables and minimize heterogeneity, it was ensured that fetal samples were collected within 1 year (June 5th, 2022‐May 11th, 2023) and were processed under strict, standardized protocols to minimize technical and biological variability. Immediately after collection, brain samples were quickly dissected from the STP region, washed with 1× PBS, and then cryopreserved in liquid nitrogen until further processing. All tissue samples were handled identically and stored in liquid nitrogen. In addition, samples from both NSCLP and Con groups underwent the same RNA sequencing protocol to avoid technical biases in transcriptomic profiling.

For RT‐qPCR detection, the brain tissue was directly frozen at −80 °C. For immunofluorescence staining detection, the brain tissues were fixed in paraformaldehyde. The tissue was then processed using a series of sucrose gradients and stored in optimal cutting temperature (OCT) compound for embedding. Samples were sectioned at 15 µm thickness using a cryostat and stored at −20 °C for further analysis.

### Nuclei Isolation and Capture

Mechanical extraction method was applied to separate nuclei. Dounce pestle A was utilized to grind tissues 10 times and ground with Dounce pestle B 10 times. The tissues were put into 2 mL Dounce homogenizer set as well as thawed in homogenization buffer including 500 mMm sucrose, 0.1% NP‐40, 1% bovine serum albumin, 20 mMm Tris pH 8.0, 1× protease inhibitor cocktail, 0.2U µL^−1^ RNase inhibitor, and 0.1 mMm DTT. The specimens were then passed through a 30‐µm cell strainer and centrifuged for 5 min at 500×g at 4 °C to pellet the nuclei. Finally, the nuclei were resuspended in the wash buffer. After washing, the nuclei were resuspended in 1 mL of wash buffer, centrifuged again at 500 × g for 5 min and resuspended with Cell Resuspension Buffer.

### RNA Library Construction and Sequencing

snRNA‐seq were performed by the DNBelab C4 platform. DNBelab C Series High‐throughput Single‐Cell RNA Library was applied for snRNA‐seq library preparation as previously described.^[^ ^47^ ^]^ Briefly, single‐cell suspensions were used to generate barcoded snRNA‐seq libraries by droplet generation, emulsion breakage, bead collection, reverse transcription, as well as cDNA amplification. Indexed sequencing libraries having short cDNA fragments with 250–400 bp were constructed according to the manufacturer's protocol. After qualification using the Qubit ssDNA Assay Kit (Thermo Fisher Scientific). All libraries were further sequenced by the MGISEQ‐2000 sequencing platform. The sequencing reads included 30 bp for read 1 consisting of 10‐bp cell barcode 1, 10‐bp cell barcode 2 and 10‐bp unique molecular identifiers (UMI), 100‐bp read 2 for transcript sequences, as well as 10‐bp barcodes read for sample index.

### Sequence Alignment and Data Transformation

Raw reads were preprocessed using the open‐source pipeline (https://github.com/MGI‐tech bioinformatics/ DNBelab_C_Series_snRNA‐analysis‐software) with default parameters. Processed reads were aligned to the GRCh38 reference genome by STAR‐2.7.11 b.^[^ ^48^ ^]^ Valid cells were identified according to the UMI number distribution of each cell using the “barcodeRanks” function of the DropletUtils tool. Background beads and the beads with low‐quality UMI counts were removed. Finally, the gene expression of cells was calculated by applying PISA, and a gene x cell matrix for each library was generated.

### Data Quality Control and Processing

R package Seurat v.4.4 was utilized to quality control as well as downstream analyses. For each sample, genes expressed in < 10 cells were removed. Cells with a percentage of mitochondrial genes < 5%, UMI counts of 500–40000 per cell, and gene counts of 500–6000 per cell were applied for further analyses. The counts for all cells were normalized using library size, multiplied by the default scale factor (10 000) and log transformed. The top 2000 variable genes were determined using the “FindVariableFeatures” function in Seurat. The top principal components were further applied for dimensionality reduction, clustering, and visualization by t‐SNE orUMAP.

### Identification of Main Cell Types and Subtypes

Marker genes for each cluster were identified with MAST test in the “FindAllMarkers” function by the cutoff criteria, including Benjamini‐Hochberg adjusted *p* value < 0.05, the log fold change > 0.50, and genes being detected in > 0.20 of the cells for their corresponding cluster. The following main cell types were used for cell annotation according to the Cell Taxonomy database (https://ngdc.cncb.ac.cn/celltaxonomy/) and known brain cell markers:^[^ ^19^, ^49^, ^50^, ^51^, ^52^ ^]^ ExN: NEUROD6, SATB2, and NEUROD2; InN: GAD1 and DLX6‐AS1; Astro: CST3 and AQP4; OPCs: PDGFRA, OLIG1, and COL9A1; EC: FN1, IGFBP7, and CLDN5; Micro: SPP1, C3, P2RY12, CSF3R, and CX3CR1; CR: RELN. The following layer‐specific markers for ExN subtypes were used:^[^ ^49^, ^52^ ^]^ layers II: GLRA3; layers II/III: CARTPT and LAMP5; layers II‐IV: THSD7A and CUX2; layers II‐VI: PVRL3 and RASGRF2; layers III/IV: PRSS12; layers IV/V: RORB; layers IV‐VI: GRIK4; layers V: SULF2, PCP4, KCNK2, HTR2C and FEZF2; layer V/VI: RPRM, RXFP1, TOX, ETV1, and FOXP2; layers VI: NR4A2, SYNPR, NTNG2, TLE, and ADRA2A.

### WGCNA Analysis in ExN and InN Subtypes

WGCNA analysis was performed in ExN and InN subtypes by R packages hdWGCNA v.0.3.00^[^ ^53^ ^]^ and WGCNA v.1.72‐5.^[^ ^54^ ^]^ First, bi‐weighted mid‐correlations were calculated for all pairs of genes to create the signed similarity matrix. The optimal soft power value was identified by the pickSoftThreshold function. We then performed network construction and module detection via the blockwiseModules function. Finally, modules were obtained utilizing specific module‐cutting parameters, containing a minModuleSize of 50 genes, the deepSplit score of 2–4, a mergeCutHeight of 0.25 and a threshold of correlation of 0.2. Modules associated with the specific cell subtype were considered as standard modules for classifying genes into the corresponding cell subtypes. For InN subtypes, 5 modules were obtained, and 7 modules were obtained in ExN subtypes.

### Differential Expression Analyses

Differential expression analysis was performed between two comparison groups using “wilcoxauc” function implemented in R package immunogenomics/presto v.1.0.0 or “FindAllMarkers” function (MAST test) in Seurat. The gene expressed in at least 0.20 of single cells in one of two groups was applied to analyzed for the corresponding cell type. Benjamini–Hochberg adjusted *p* value < 0.05, as well as the |log fold change| > 0.25 in gene expression between two groups, was regarded as the cutoff criteria for identifying DEGs.

### Machine Learning Models Identify Hub DEGs

Sixteen binary‐classification artificial intelligence algorithms containing Linear Discriminant Analysis, Ridge Classifier, Logistic Regression, CatBoost Classifier, Random Forest Classifier, Extra Trees Classifier, Extreme Gradient Boosting, Decision Tree Classifier, Gradient Boosting Classifier, Light Gradient Boosting Machine, K Neighbors Classifier, Naive Bayes, SVM‐Linear Kernel, Ada Boost Classifier, Quadratic Discriminant Analysis and Dummy Classifier were used to further identify the key DEGs between two comparison groups in each cell type. First, the count matrices for the corresponding DEGs and classified labels (0 or 1) were extracted from Seurat and used to build a labeled data matrix. After filtering out lowly expressed genes, normalizing the expression values, and addressing any batch effects or technical biases, the area under the receiver‐operating‐characteristic (ROC), precision‐recall (PR) curve, Accuracy, Recall, Prec and F1 score of 16 artificial intelligence algorithms were assessed and the optimal model was determined to conduct on the further analyses based on the highest accuracy of all methods tested. The pretreated data were divided, with 80% utilized for training (internal validation), to avoid problems with overfitting. The other 20% data set was used for testing (external validation). To ensure reliable results, 10‐fold cross‐validations in the training of the prediction model were conducted. Finally, Recursive Feature Selection method was utilized to identify the key DEGs associated with the group classified label 1.

### The Hub Regulatory Network Identification for TFs and Their Target Genes

SCENIC was utilized to identify the hub TFs and their target genes within each cell type by pySCENIC v.0.12.1.^[^ ^55^ ^]^ Briefly, the lists of 1892 TFs (allTFs_hg38.txt), Motif2TF annotations (motifs‐v9‐nr.hgnc‐m0.001‐o0.0.tbl), motif data (hg38_refseqr80_10kb_up_and_down_tss.mc9nr.genes_versus_motifs and hg38_refseqr80_500bp_up_and_100bp_down_tss.mc9nr.genes_versus_motifs) from the cisTarget resources website (https://resources.aertslab.org/cistarget/) were obtained.The indirect targets were then pruned from above modules through cis‐regulatory motif discovery. AUCell enrichment score was finally applied to assess activity of those regulons in cells. The results were visualized in R package SCENIC v.1.3.1.^[^ ^56^ ^]^ TF‐targets networks were constructed by Cytoscape v.3.10.2 and Java 17.^[^ ^57^ ^]^

### Functional Enrichment Analyses

Functional enrichment analyses including GO and KEGGwere performed for cell marker genes and DEGs with R packages msigdbr v7.5.1, clusterProfiler v4.2.2, and org.Mm.eg.db v3.16.0.

### Cell‐Cell Communication Analysis

CellChat v.2.0, a tool to quantitatively infer intercellular communication networks from snRNA‐seq data,^[^ ^59^ ^]^ was applied to evaluate cell‐cell communication. The analysis processes referred to the CellChat guide (http://www.cellchat.org/). The difference of connection number and strength and distribution of signaling pathways between two comparison groups was compared.

### Pseudotime Trajectory Analysis

The R package Monocle 3 (v.1.3.5) was applied to construct pseudo‐temporal trajectories of ExN and InN subtypes and explore the dynamics of gene expression within those cell subtypes over time.^[^ ^60^ ^]^ First, the normalized expression values were used to reduce the dimensionality of the data by UMAP. Subsequently, the origin of cells was determined using the “order_cells” function. Finally, the trajectory was visualized using the “plot_cells” and “plot_cell_trajectory” functions. The R package Monocle 2 (v.2.22)^[^ ^61^ ^]^ was used to show the dynamics of gene expression within main cell types between different groups. For Monocle 2 analysis of gene expression, the highly variable genes were used to sort cells into a pseudotime order. The “reduceDimension” function was applied to reduce dimension. Significant genes were determined using the differentialGeneTest function, with *q*‐value < 0.001.

### Real‐Time Quantitative Polymerase Chain Reaction (RT‐qPCR)

Total RNA was extracted from cells or tissues using TRIzol reagent (Invitrogen, USA), and RNA concentration and purity were assessed spectrophotometrically. First‐strand cDNA was synthesized from total RNA with the PrimeScript RT Reagent Kit (Takara, Japan) following the manufacturer's instructions. Gene‐specific primers were designed and synthesized by Akihiko Biological Company with the following sequences: MEF2C‐F (GGCAACAGCAACACACCTACATAACATG) and MEF2C‐R (GAGTAGAAGGCAGGGAGAGAGATTTGAAC); ZBTB38‐F (ATGATGGCAGTTCACCTGGTAACACAC) and ZBTB38‐R (GCTGAATCCGAATCCTGTGGTATGG); CREB1‐F (ATTCACAGGAGTCAGTGGATAGT) and CREB1‐R (CACCGTTACAGTGGTGATGG). Each 20 µL qPCR reaction system contained 10 µL SYBR Green Master Mix (Vazyme, China), 1 µL cDNA template, 0.8 µL of each primer (10 µMm), and 7.4 µL nuclease‐free water. Amplification was performed on a real‐time PCR instrument under the following cycling conditions: initial denaturation at 95 °C for 5 min, followed by 40 cycles of 95 °C for 10 s, 60 °C for 15 s, and 72 °C for 20 s. Melting‐curve analysis confirmed amplification specificity. Relative gene expression was calculated using the 2^−ΔΔCt^ method, with β‐actin serving as the internal reference.

### Immunofluorescence Staining

Frozen brain sections were removed from −20 °C storage, allowed to equilibrate to room temperature, and baked at 37 °C for 10 min to prevent tissue detachment. Sections were washed 3 times with PBST (5 min each). Antigen retrieval was performed in 1× sodium citrate–EDTA buffer (diluted from 40× stock) preheated to 95 °C, by boiling the slides in a pressure cooker (800 W) for 20 min. After cooling to room temperature, excess liquid was removed, the tissue perimeter was circled with a hydrophobic barrier pen, and sections were washed 3 times again with PBST (3 min each). Sections were blocked with sealing buffer at room temperature for 3 hours, then incubated overnight (16–18 h) at 4 °C with primary antibodies diluted in 2% sheep serum: MEF2C (Abcam, ab211493, rabbit), SYN (Abcam, ab32127, rabbit), and PSD95 (Abcam, ab18258, rabbit). After 5 PBST washes (5 min each), slides were incubated for 3 hours at room temperature in the dark with HRP‐conjugated secondary antibody (Meyers, kit‐5020) diluted in PBS, followed by three PBST washes (5 min each).

Fluorescent labeling was achieved with TYR‐488 and TYR‐555 dyes for 10 min. Nuclei were counterstained with DAPI (1:3000 in PBS) for 10 min in the dark, then washed 3 times with PBST (5 min each). Slides were mounted in glycerol and scanned using a whole‐slide scanner. Fluorescence images were captured with a Nikon AX confocal microscope using 20× and 60× objectives and NIS‐Elements AX software (image size 2048 × 2048 pixels). Fiji was used for cell counting quantification.

### Lentiviral Construction and Selection of MEF2C shRNA

Three lentiviral vectors carrying shRNAs targeting rat MEF2C (LV‐Mef2c‐RNAi‐P25G1215, −1216, −1217) and a negative control lentivirus (CON313) were obtained (titers: 8.60E+08TU/mL, 1.10E+09TU/mL, 1.00E+09TU/mL, and 7.60E+08TU/mL, respectively). HEK293T cells were cultured in DMEM/F12 supplemented with 10% fetal bovine serum (FBS) and 1% penicillin–streptomycin. When cells reached 80–90% confluence, they were seeded into six‐well plates (2×10^5^ cells/well) and transduced with lentivirus at a multiplicity of infection (MOI) of 1, using 25× transfection reagent (40 µL/well). Viral medium was replaced after 12 hours, and fluorescence was evaluated 72 hours post‐transduction. RNA was extracted with TRIzol, reverse transcribed, and subjected to RT‐qPCR to evaluate MEF2C knockdown efficiency, using GAPDH as a control. The shRNA construct producing the greatest reduction in MEF2C expression was selected for subsequent experiments.

### Primary Cortical Neuron Culture and Lentiviral Transduction

Primary cortical neurons were prepared from postnatal day 1 Sprague–Dawley rats. Poly‐D‐lysine (PDL)‐coated coverslips (50 µg mL^−1^, incubated overnight at 37 °C) were prepared in advance. Following decapitation, cortical tissue was dissected on ice, meninges and vasculature removed under a stereomicroscope, and tissue transferred to ice‐cold DMEM/F12 with antibiotics. Cortices were minced, digested in 0.25% trypsin (optionally with DNase) at 37 °C for 15 min, and gently triturated with a fire‐polished Pasteur pipette. The cell suspension was filtered through a 70 µm strainer and centrifuged at 1000 rpm for 8 min. Cells were plated at 5×10^5^–1×10^6^ cells/well in six‐well plates containing PDL‐coated coverslips and complete medium (DMEM high glucose + 10% FBS + 1% penicillin–streptomycin). After 4 hours, the medium was replaced with Neurobasal‐A medium supplemented with B‐27, GlutaMAX, and antibiotics. Half‐medium changes were performed every 3 days.

The cultured neurons were transduced with MEF2C shRNA lentivirus (LV‐MEF2C‐RNAi‐P25G1215) or control virus at an MOI of 5. Virus was added to antibiotic‐free Neurobasal‐A medium (1 mL/well) with 25× transfection reagent (40 µL/well). After 12 hours, the medium was completely replaced. Transduction efficiency was assessed at 72 hours post‐infection by fluorescence microscopy. Neurons were maintained until day 7 for downstream analyses.

### Immunofluorescence Staining In Vitro

At culturing day 7, neuronal cultures were fixed with 4% paraformaldehyde for 20 min, washed three times in PBS, and permeabilized/blocked with 5% goat serum containing 0.3% Triton X‐100 for 2 hours at room temperature. Primary antibodies against Tuj1 (Abcam, ab78078, Mouse), PSD95 (Abcam, ab18258, rabbit), SYN (Abcam, ab32127, rabbit), and MEF2C (Abcam, ab211493, rabbit) were applied overnight at 4 °C (2% goat serum dilution). After washing, species‐specific Alexa Fluor–conjugated secondary antibodies and DAPI were applied. Coverslips were mounted on glass slides and imaged under a fluorescence microscope. Groups included untreated control (Normal), negative control (shNC), and MEF2C knockdown (shMEF2C). Fiji (ImageJ, NIH) was used for quantitative image analysis. Maximum intensity projections of the acquired z‐stacks were generated, and axonal length, dendritic spine density, synaptic puncta number, and fluorescence intensity were quantified. Dendritic spines and synapses were manually traced in the GFP channel, and corresponding ROIs were transferred to other channels to measure mean intensity (after background subtraction) and spine area. For each group, five coverslips were analyzed, and three neurons were quantified from five randomly selected fields per coverslip. Data are presented as mean ± SD, and statistical significance was determined using Student's *t*‐test or ANOVA.

### Primary Cortical Neuron Culture and Patch‐Clamp Recordings

Primary cortical neurons were isolated from embryonic day 18 (E18) Sprague–Dawley rat embryos. Following enzymatic dissociation, cells were plated onto precoated glass coverslips and cultured in six‐well plates until day 10 for electrophysiological recordings. Coverslips were incubated at 32 °C in bicarbonate‐buffered artificial cerebrospinal fluid (aCSF) equilibrated with a 95% O_2_/5% CO_2_ gas mixture. The aCSF was composed of 126 mMm NaCl, 26 mMm NaHCO_3_, 10 mMm glucose, 2.5 mMm KCl, 1.25 mMm NaH_2_PO_4_, 1 mMm MgSO_4_, and 2 mMm CaCl_2_. Fluorescently labeled neurons were visualized under an upright BX51W1 fluorescence microscope (Olympus). Recording pipettes were pulled from borosilicate glass (resistance: 4–9 MΩ when filled) and filled with a low‐chloride internal solution containing 145 mMm K⁺ gluconate, 0.1 mMm CaCl_2_, 2.5 mMm MgCl_2_, 10 mMm HEPES, 0.2 mMm EGTA, and 4 mMm Na⁺ phosphocreatine. Whole‐cell recordings were performed using a MultiClamp 700B amplifier and digitized with a Digidata 1550B converter (Molecular Devices).Data were acquired with Clampex 10.7 software (Molecular Devices) and low‐pass filtered at 10 kHz. Action potential properties were analyzed in Clampfit 10.7.0.3, while sEPSCs were analyzed using MiniAnalysis 6.0.7 (Synaptosoft).

### Statistical Analysis

All statistical analyses were performed using SPSS v.26.0 (IBM, USA) and GraphPad Prism 9.0 (GraphPad Software, USA). Quantitative data were first assessed for normality by Shapiro–Wilk test. Normally distributed data are presented as mean ± SEM/SD, while non‐normally distributed data are presented as median with interquartile range (IQR) where applicable. The sample size (*n*) for each analysis is provided in the corresponding figure legends. Each *n* refers to a biological replicate (individual fetal samples). For comparisons between two groups, the independent sample *t*‐test was used for normally distributed data, and the nonparametric test (Mann–Whitney test) was used for nonnormally distributed data. For comparisons among three groups, one‐way ANOVA was applied. For correlation analysis, Pearson correlation was applied when the data conformed to the normal distribution, and Spearman correlation was used when the data did not meet the normal distribution. All statistical tests were two‐sided, and *p* value less than 0.05 was considered statistically significant. For RT‐qPCR data involving multiple gene targets, Benjamini–Hochberg correction was applied to adjust for multiple comparisons.

## Conflict of Interest

The authors declare no conflict of interest.

## Author Contributions

L.‐L.X., X.‐W.H., and Q.‐X.X. contributed equally to this work. L.L.X. and T.H.W. conceived and supervised this study. X.H.T. supervised and reviewed the revised manuscript. X.W.H., Q.X.X., and Y.G.Z. performed snRNA‐seq data analysis. H.Y.Q. wrote the original manuscript. L.C. revised the manuscript and contributed to graphmaking. C.L.F. and Q.L.W. contributed to data analyses and reviewed the manuscript. L.R.H. and R.L.D. performed validation experiments. Y.Q.F., Y.Y.Z., and C.Y.Z. contributed to collecting clinical samples. Z.M.C., S.K.Y., Y.J.Z., X.M.H., and T.T.Z. were involved in the quantification of immunofluorescence results. All authors discussed the results and approved the manuscript.

## Supporting information



Supporting Information

Supplemental Tables 1‐4

## Data Availability

The raw sequence data that support the findings of this study are openly available in Genome Sequence Archive in National Genomics Data Center (https://ngdc.cncb.ac.cn/gsa‐human) under data accession number HRA012374.
