# Supplementary material for: Single‐Nucleus RNA Sequencing Reveals Mid‐Gestational Neurodevelopment Features in the Superior Temporal Plane from Fetuses with Nonsyndromic Cleft Lip and Palate
Source: Adv Sci (Weinh). 2025 Oct 21;13(1):e04191. doi: 10.1002/advs.202504191 (PMC12767050; doi:10.1002/advs.202504191)
Supplement: Supplementary file 1 — Supporting Information [file ADVS-13-e04191-s001.pdf]

Supplemental figures

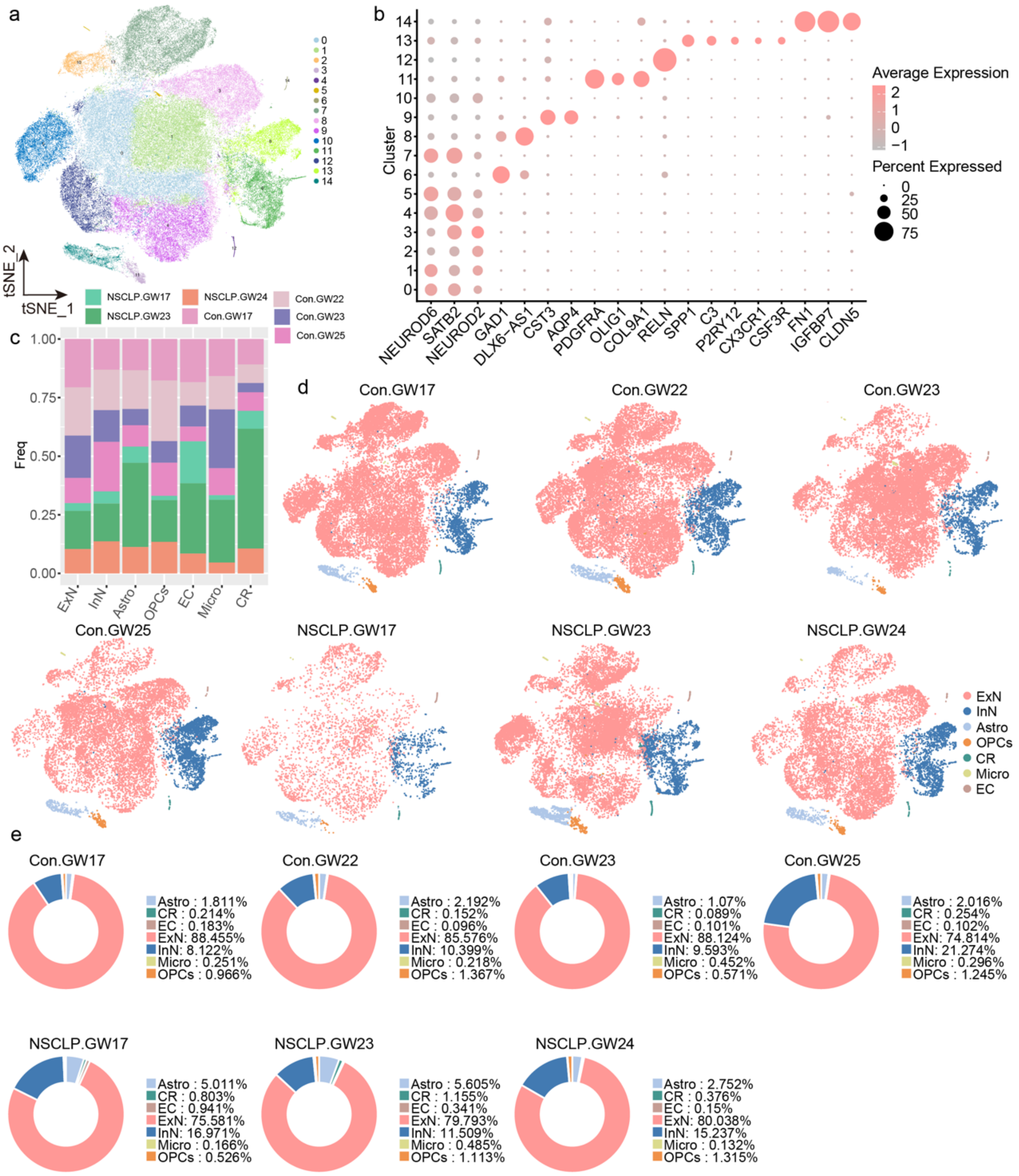

Figure S1. Single-nucleus transcriptomic landscape of seven fetal STP samples. a) t-SNE projections illustrate cell clusters and group assignments across all samples (*n* = 5 fetuses for Con; *n* = 4 fetuses for NSCLP. b) Dot plot of average expression and detection frequency of representative marker genes across cell types. c) Stacked bar charts showing proportions of major cell types per sample in control and NSCLP groups. d) t-SNE projections illustrate sample-specific cell distributions. e) Percentage distribution of cell types at different gestational weeks between groups.

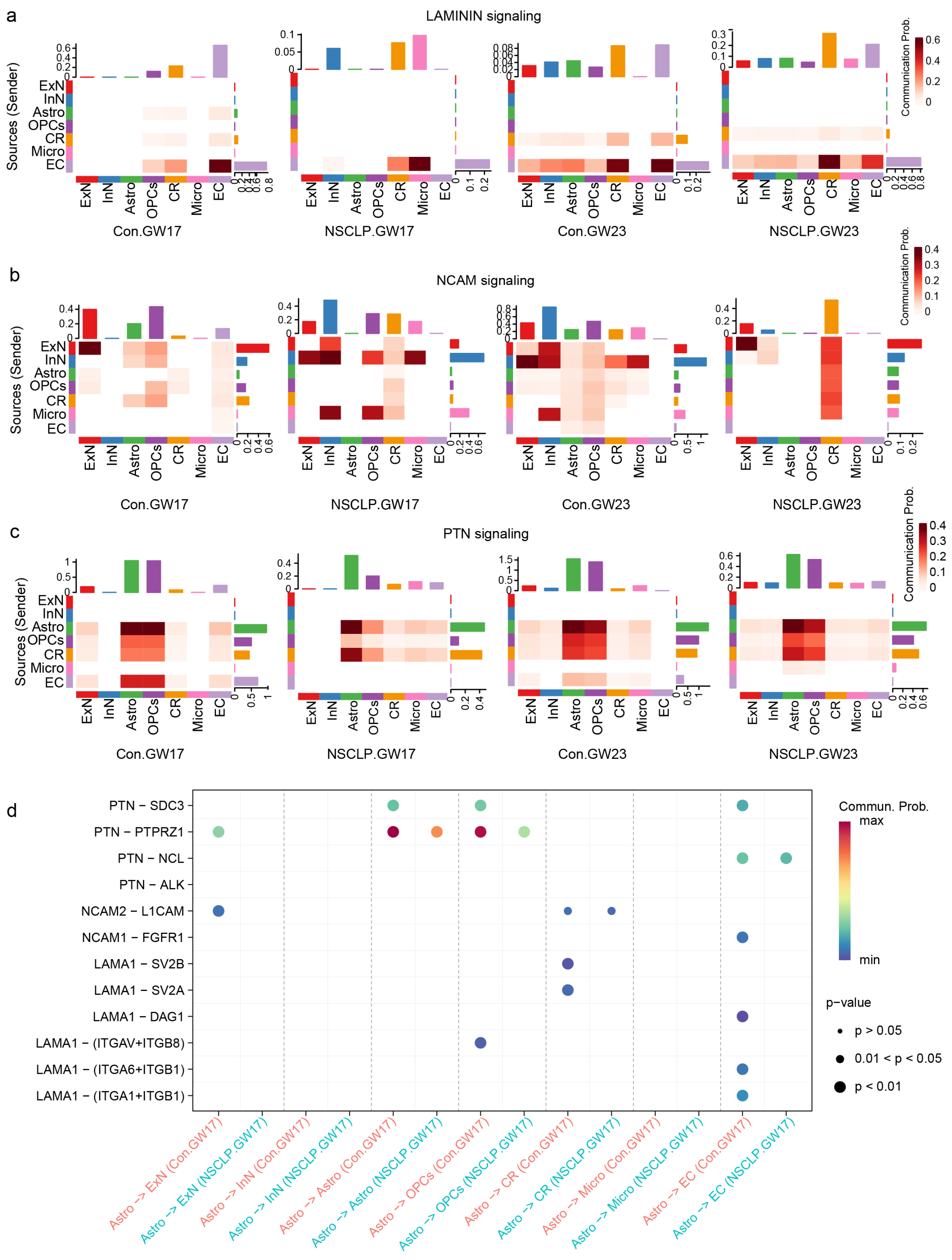

Figure S2. Differential LAMININ, NCAM and PTN signaling across cell types. a-c) Cell–cell communication intensity of the LAMININ, NCAM and PTN pathways in seven major cell types comparing control and NSCLP fetuses at GW17 and GW23. d) Communication probability of key ligand–receptor pairs within these pathways between astrocytes and other cell types at GW17. Interaction strength was calculated using CellChat with permutation tests (BH-adjusted $p < 0.05$).

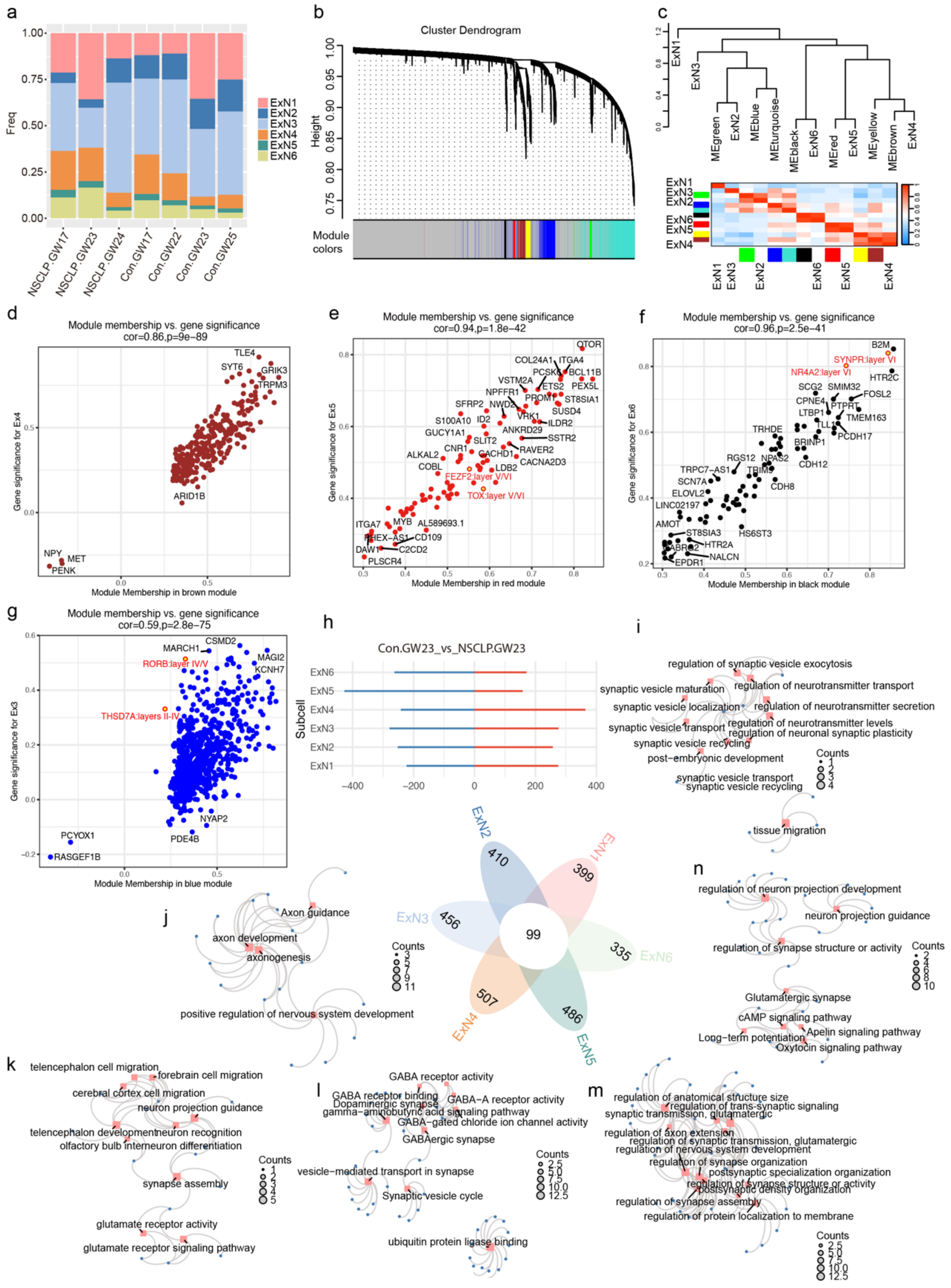

Figure S3. ExN subtype alterations and gene-module associations. a) Stacked bar plot of ExN subtype proportions between control and NSCLP groups stratified by gestational week. b) Hierarchical clustering dendrogram of ExN cells. c) Heatmap of correlations between weighted gene co-expression network analysis (WGCNA) modules and ExN subtypes. d–g) Scatter plots showing module-membership vs. gene-

significance correlations for key subtypes. h) Numbers of dysregulated genes at GW23 (BH-adjusted $p < 0.05$, $|\log_2 FC| > 0.25$). i–n) GO function enrichment analysis of subtype-specific differentially expressed genes.

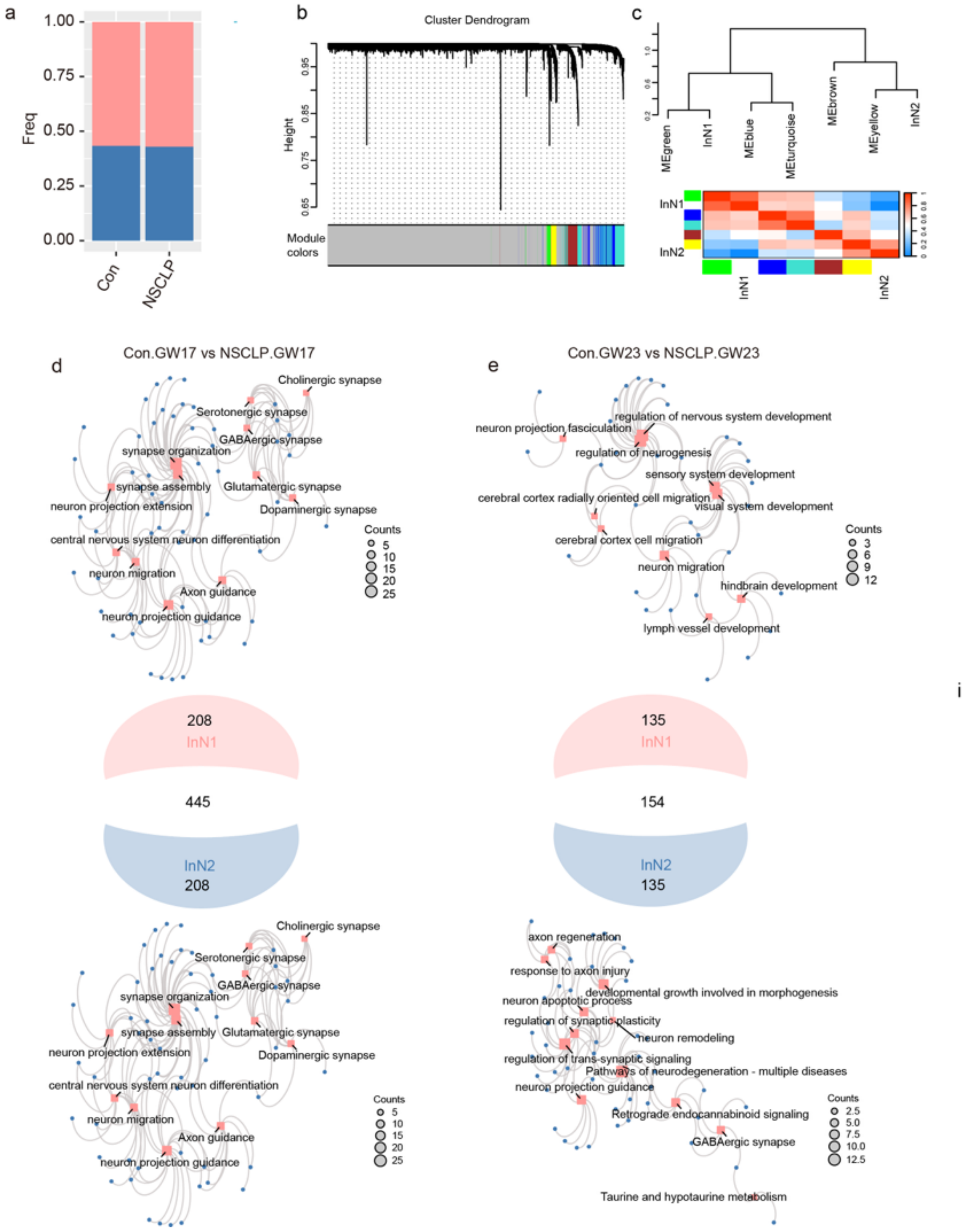

Figure S4. InN subtype analysis. a) Stacked bar plot of InN subtype proportions across groups and gestational weeks. b) Clustering dendrogram of InN cells. c) Heatmap shows the correlation of InN subtypes with WGCNA modules. d–e) GO functional

enrichment of dysregulated genes in InN1 and InN2 subtypes at GW17 and GW23 (BH-adjusted $p < 0.05$).

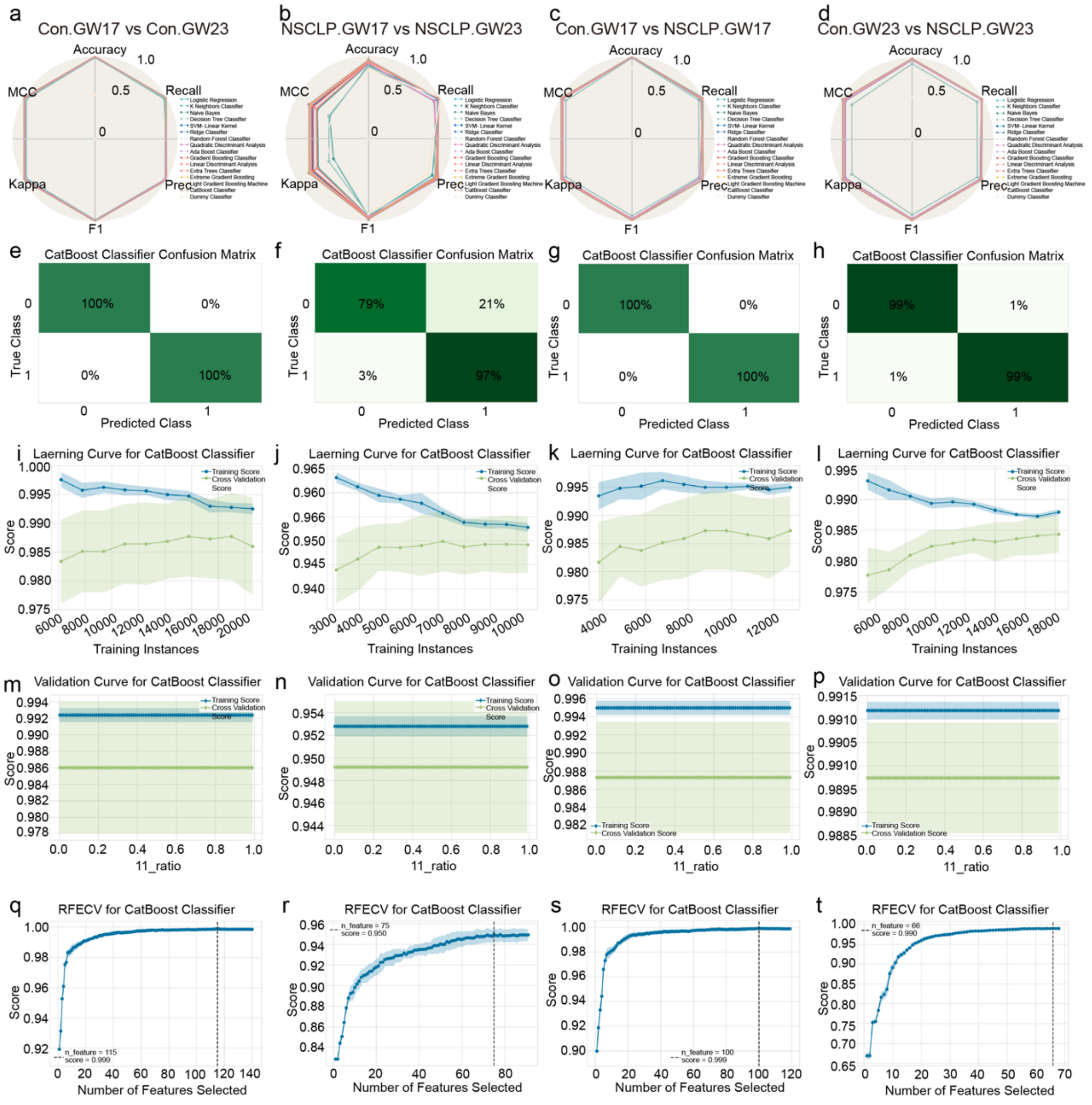

Figure S5. Machine-learning classification of ExN developmental states. a–d) Radar charts of CatBoost classifier performance for pairwise group comparisons (Con.GW17 vs. Con.GW23, NSCLP.GW17 vs. NSCLP.GW23, Con.GW17 vs. NSCLP.GW17, Con.GW23 vs. NSCLP.GW23). e-h) Confusion matrix of CatBoost classifier for pairwise group comparisons. i-l) The learning curve of CatBoost classifier for pairwise group comparisons. m-p) Validation curve of CatBoost classifier for pairwise group comparisons. q-t) Recursive feature elimination cross validation (RFECV) curve of CatBoost classifier for pairwise group comparisons. Performance metrics represent mean ± SD of 10-fold cross-validation.

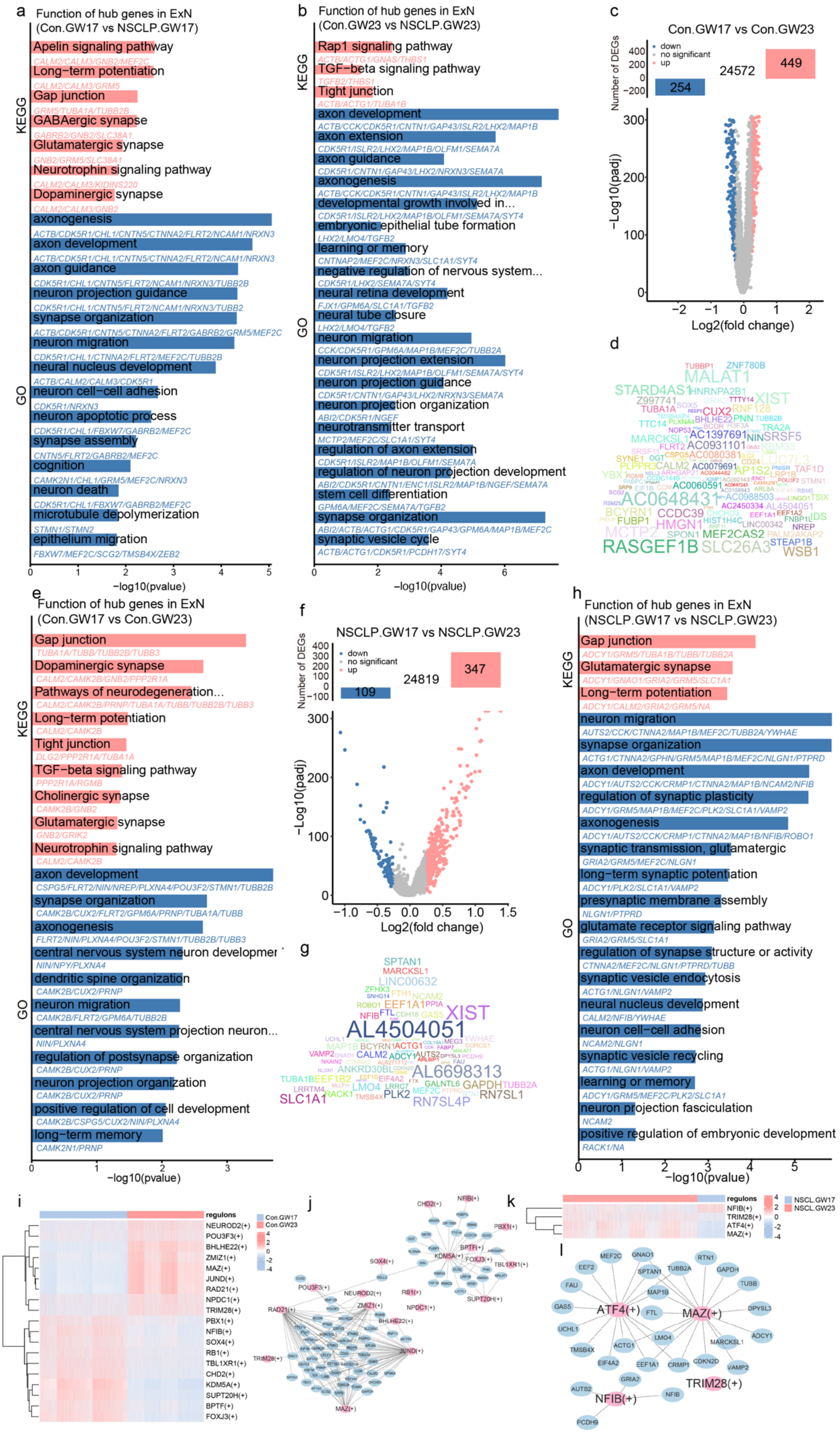

a
Function of hub genes in ExN
(Con.GW17 vs NSCLP.GW17)
KEGG
Apelin signaling pathway
Long−term potentiation
Gap junction
GABAergic synapse
Glutamatergic synapse
Neurotrophin signaling pathway
Dopaminergic synapse
GO
axonogenesis
axon development
axon guidance
neuron projection guidance
synapse organization
neuron migration
neural nucleus development
neuron cell−cell adhesion
neuron apoptotic process
synapse assembly
cognition
neuron death
microtubule depolymerization
epithelium migration
−log10(pvalue)
b
Function of hub genes in ExN
(Con.GW23 vs NSCLP.GW23)
KEGG
Rap1 signaling pathway
TGF−beta signaling pathway
Tight junction
GO
axon development
axon extension
axon guidance
axonogenesis
developmental growth involved in...
embryonic epithelial tube formation
learning or memory
negative regulation of nervous system...
neural retina development
neural tube closure
neuron migration
neuron projection extension
neuron projection guidance
neuron projection organization
neurotransmitter transport
regulation of axon extension
regulation of neuron projection development
stem cell differentiation
synapse organization
synaptic vesicle cycle
−log10(pvalue)
c
Con.GW17 vs Con.GW23
Number of DEGs
down
no significant
up
254
24572
449
−Log10(padj)
Log2(fold change)
d
e
Function of hub genes in ExN
(Con.GW17 vs Con.GW23)
KEGG
Gap junction
Dopaminergic synapse
Pathways of neurodegeneration...
Long−term potentiation
Tight junction
TGF−beta signaling pathway
Cholinergic synapse
Glutamatergic synapse
Neurotrophin signaling pathway
GO
axon development
synapse organization
axonogenesis
central nervous system neuron development
dendritic spine organization
neuron migration
central nervous system projection neuron...
regulation of postsynapse organization
neuron projection organization
positive regulation of cell development
long−term memory
−log10(pvalue)
f
NSCLP.GW17 vs NSCLP.GW23
Number of DEGs
down
no significant
up
109
24819
347
−Log10(padj)
Log2(fold change)
g
h
Function of hub genes in ExN
(NSCLP.GW17 vs NSCLP.GW23)
KEGG
Gap junction
Glutamatergic synapse
Long−term potentiation
GO
neuron migration
synapse organization
axon development
regulation of synaptic plasticity
axonogenesis
synaptic transmission, glutamatergic
long−term synaptic potentiation
presynaptic membrane assembly
glutamate receptor signaling pathway
regulation of synapse structure or activity
synaptic vesicle endocytosis
neural nucleus development
neuron cell−cell adhesion
synaptic vesicle recycling
learning or memory
neuron projection fasciculation
positive regulation of embryonic development
−log10(pvalue)
i
regulons
Con.GW17
Con.GW23
j
k
regulons
NSCLP.GW17
NSCLP.GW23
l

Figure S6. Machine learning–derived hub genes and pathways in ExN. a–b) KEGG and GO pathway enrichment of ExN hub genes comparing Con vs. NSCLP at GW17 and GW23 (BH-adjusted $p < 0.05$). c) Volcano plots of differential gene expression between developmental stages within Con groups. d) Word-clouds of top-ranked hub genes of ExN in Con group. e) Pathway enrichment of hub genes in ExN at GW17 and GW23 in Con group. f) Volcano plots of differential gene expression between developmental stages within NSCLP groups. g) Word-clouds of top-ranked hub genes of ExN in NSCLP group. h) Pathway enrichment of hub genes in ExN at GW17 and GW23 in NSCLP group. i–l) Heatmaps and SCENIC regulatory networks of key transcription factors and their targets in Con and NSCLP ExN cells.

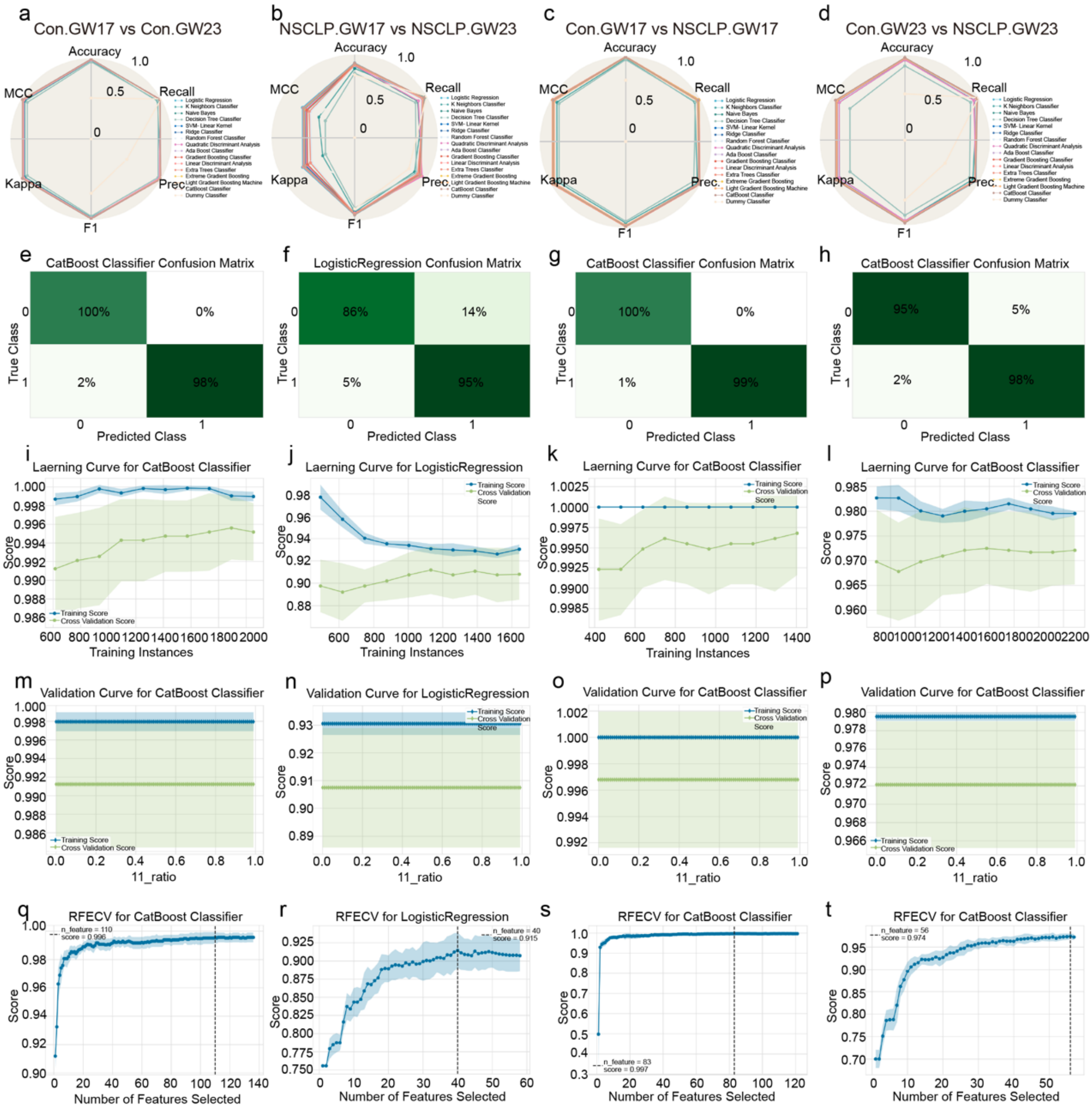

Figure S7. Machine learning classification of InN developmental states. a–d) Radar

charts of CatBoost classifier performance for pairwise group comparisons (Con.GW17 vs. Con.GW23, NSCLP.GW17 vs. NSCLP.GW23, Con.GW17 vs. NSCLP.GW17, Con.GW23 vs. NSCLP.GW23). e-h) Confusion matrix of CatBoost classifier for pairwise group comparisons. i-l) The learning curve of CatBoost classifier for pairwise group comparisons. m-p) Validation curve of CatBoost classifier for pairwise group comparisons. q-t) Recursive feature elimination cross validation (RFECV) curve of CatBoost classifier for pairwise group comparisons. Performance metrics represent mean ± SD of 10-fold cross-validation.

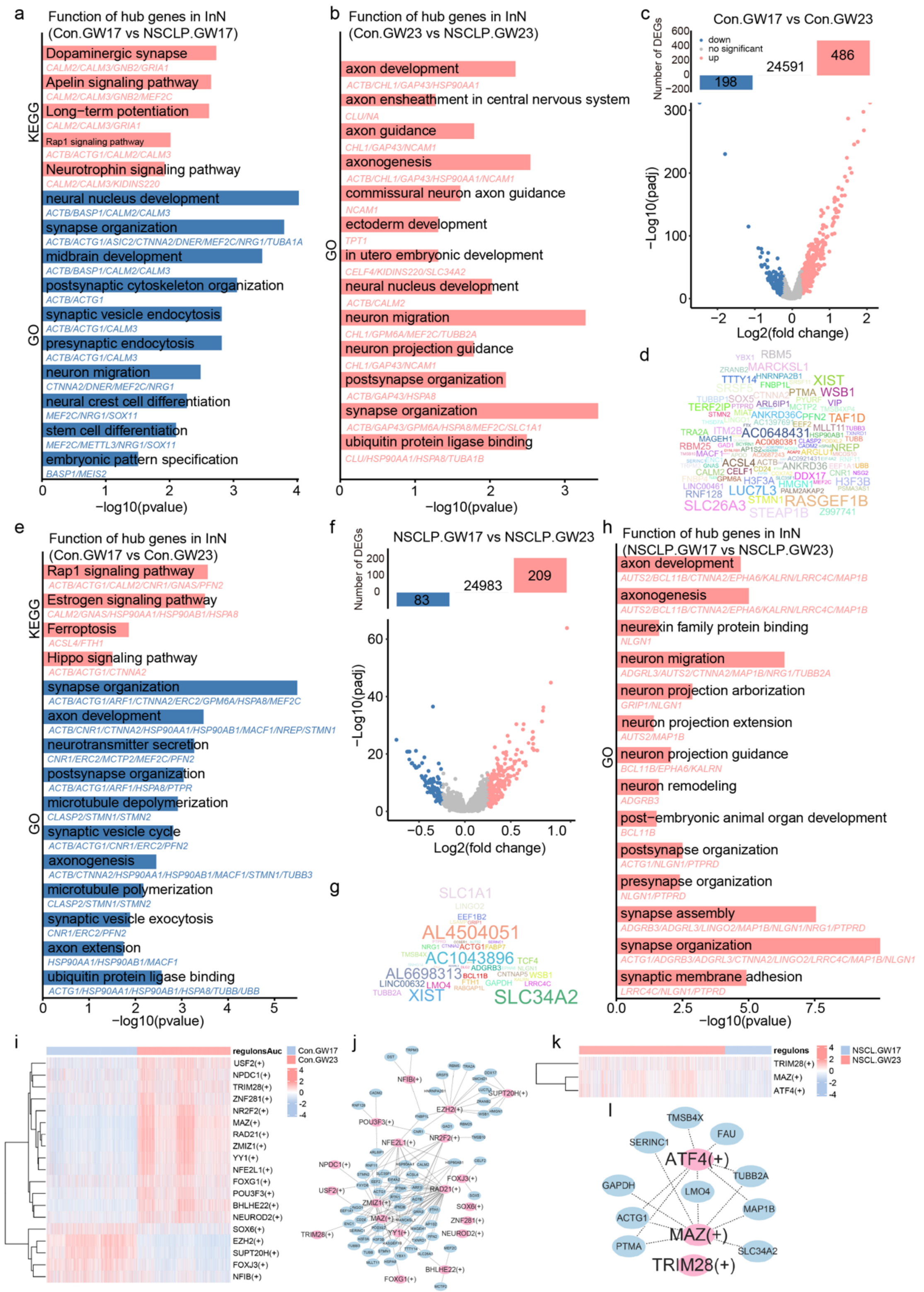

Figure S8. Machine learning analysis results of InN. a-b) KEGG and GO pathway enrichment of InN hub genes comparing Con vs NSCLP at GW17 and GW23 (BH-adjusted $p < 0.05$). c) Volcano diagram of gene expression difference in Con group at GW17 and GW23. d) Word-clouds of InN hub genes in Con group. e) Pathway

enrichment of hub genes in InN at GW17 and GW23 in the Con group. f) Volcano plots of developmental-stage differential expression in NSCLP groups. g) Word-clouds of InN hub genes in NSCLP group. h) Pathway enrichment of hub genes in InN at GW17 and GW23 in NSCLP group. i-l) Heatmaps and SCENIC-inferred TF–target regulatory networks in Con and NSCLP InN cells.

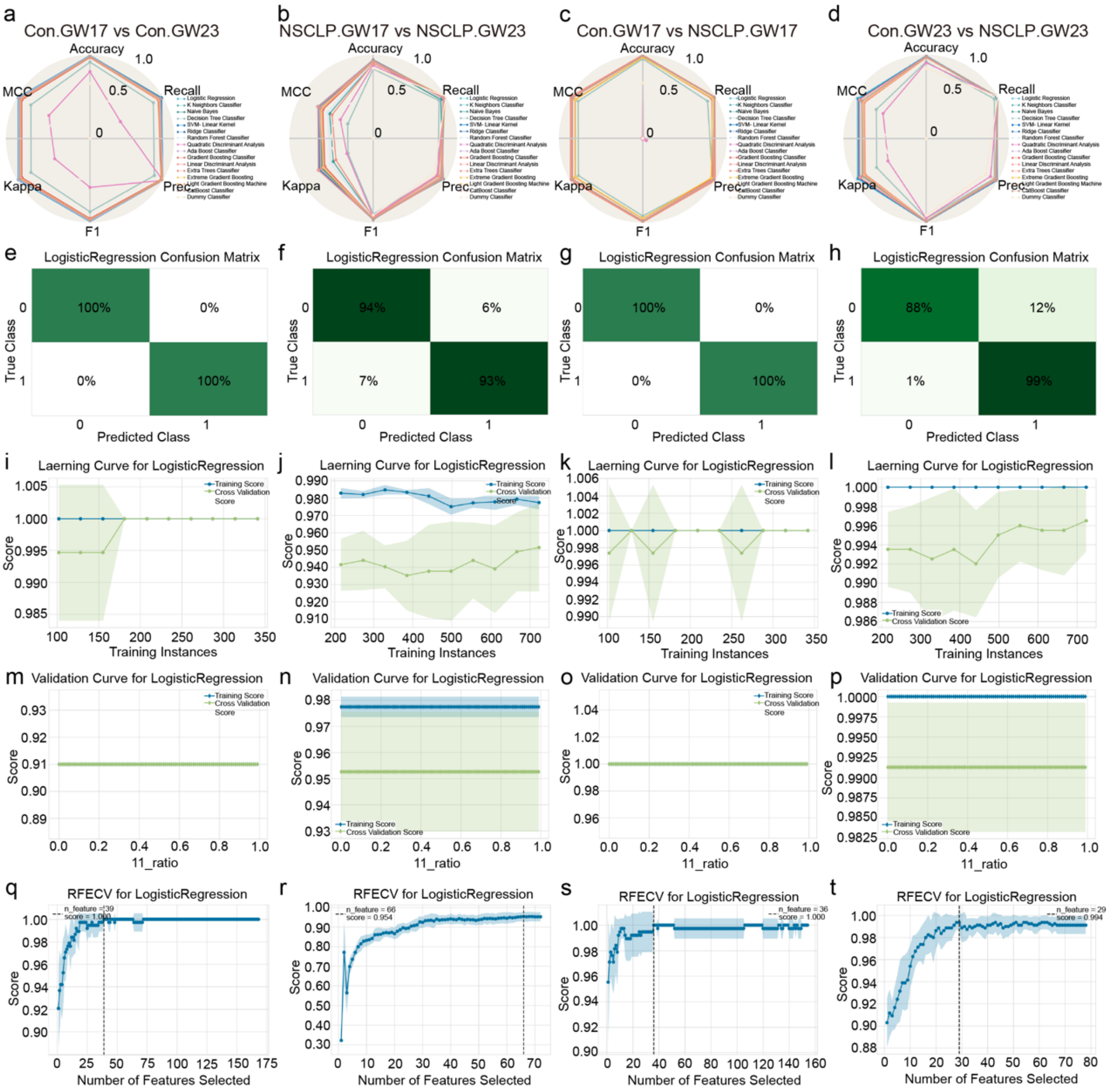

Figure S9. Machine learning classification of Astro developmental states. a–d) Radar charts of CatBoost classifier performance for pairwise group comparisons (Con.GW17 vs. Con.GW23, NSCLP.GW17 vs. NSCLP.GW23, Con.GW17 vs. NSCLP.GW17, Con.GW23 vs. NSCLP.GW23). e-h) Confusion matrix of CatBoost classifier for pairwise group comparisons. i-l) The learning curve of CatBoost classifier for pairwise group comparisons. m-p) Validation curve of CatBoost classifier for pairwise group comparisons. q-t) Recursive feature elimination cross validation

(RFECV) curve of CatBoost classifier for pairwise group comparisons. Performance metrics represent mean ± SD of 10-fold cross-validation.

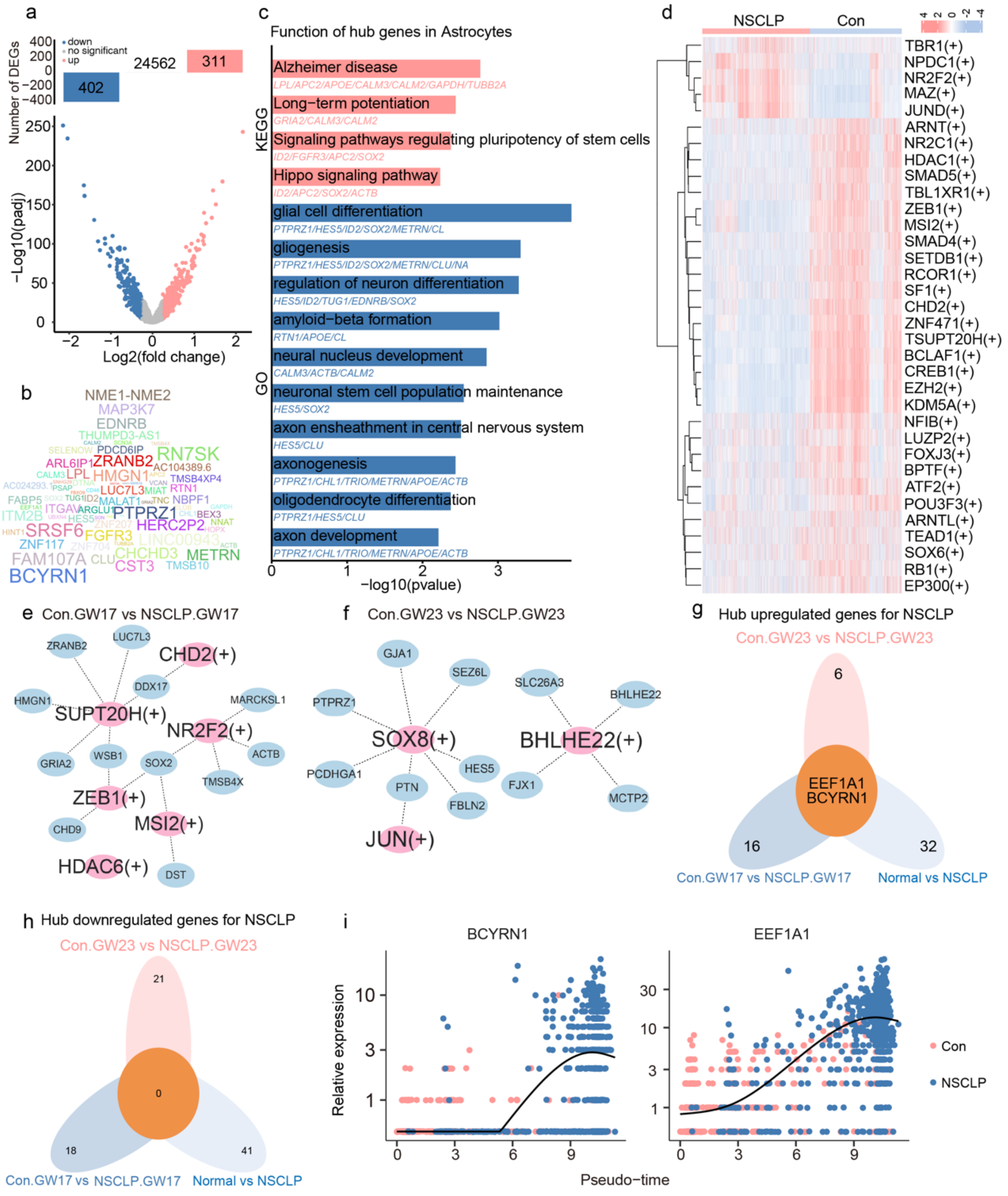

Figure S10. NSCLP-associated transcriptional networks in Astro. a) Differentially expressed genes in Astro between NSCLP and controls (BH-adjusted $p < 0.05$). b) Word-cloud of machine learning–identified hub genes related to NSCLP in Astro. c) GO enrichment of Astro hub genes related to NSCLP. d) SCENIC-identified key TFs related to NSCLP in Astro. e-f) Regulatory networks of Astro TFs and target genes at GW17 and GW23. g-h) Overlap of up- and down-regulated TFs and targets between

Con and NSCLP groups at GW17 and GW23. i) Pseudotime analysis showing dynamic expression of key TFs and targets along Astro maturation.

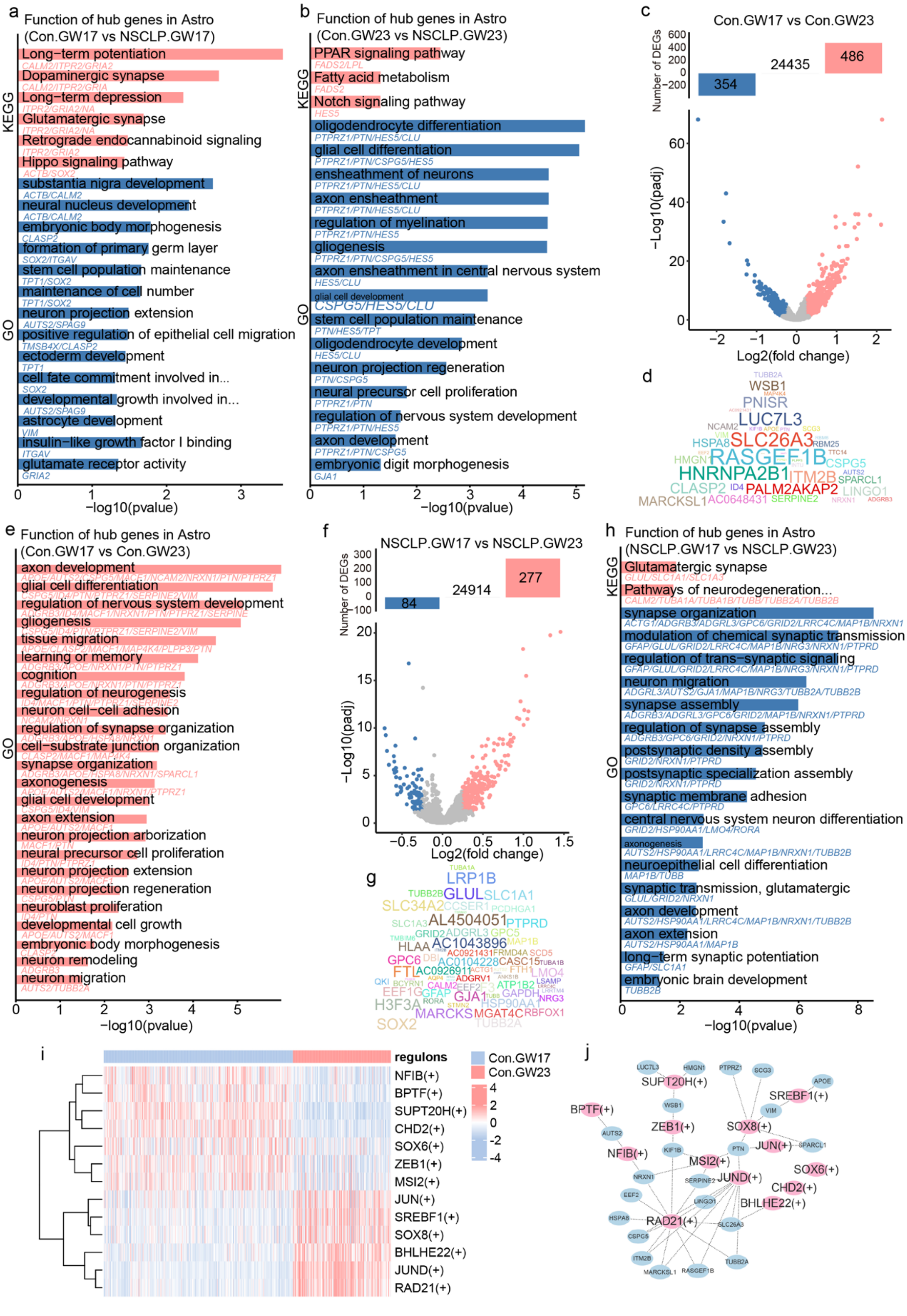

Figure S11. Machine learning and snRNA-seq analysis of Astro. a-b) KEGG and GO pathway enrichment of Astro hub genes comparing Con vs. NSCLP at GW17 and

GW23 (BH-adjusted $p < 0.05$). c) Volcano diagram of gene expression difference in Con group at GW17 and GW23. d) Word-clouds of Astro hub genes in Con group. e) Pathway enrichment of hub genes in Astro at GW17 and GW23 in the Con group. f) Volcano plots of developmental-stage differential expression in NSCLP groups. g) Word-clouds of Astro hub genes in NSCLP group. h) Pathway enrichment of hub genes in Astro at GW17 and GW23 in NSCLP group. i) Heat map of hub gene expression in Astro in Con group at GW17 and GW23. j) SCENIC-inferred TF–target regulatory networks in Con Astro cells.

Captions for supplemental tables

Table S1 Sample information for snRNA-seq data.

Table S2 Marker genes for 15 clusters.

Table S3 Functional enrichment analysis for marker gens within each cell type.

Table S4 The association between modules and hub marker genes within InN and ExN identified by WGCNA analysis.
